# Optimal placement criteria of actuators for hybrid mounting system on a non-aligned plate structure

**DOI:** 10.1038/s41598-023-44980-0

**Published:** 2023-10-18

**Authors:** Yang Qiu, Dongwoo Hong, Byeongil Kim

**Affiliations:** 1 School of Mechanical Engineering, Yeungnam University, Gyeongsan, 38541 Republic of Korea; 2 Daegu Mechatronics & Materials Institute, 32, Seongseogongdan-ro 11-gil, Dalseo-gu, 42714 Daegu, Republic of Korea

**Keywords:** Engineering, Mechanical engineering

## Abstract

Electric motors in electric and hybrid vehicles generate mid-frequency noise and vibration. These factors cause drivers to experience discomfort while traveling. Active mounting techniques have been extensively researched and developed to effectively address this issue. The optimal placement of an active mounting system is essential for enhancing NVH performance when an active mounting system is utilized. In order to propose optimal location criteria for active paths, this paper concentrates on developing an analytical model based on both dynamic and static analysis. The secondary forces along active trajectories are mathematically determined when a structure is subjected to an excitation force. These locations are considered optimal for the active mounting system if the secondary forces are comparatively minimal. Simulations and feasibility experiments are also conducted in order to validate the proposed method. In addition, the results are compared with the case of beam structure. It has been determined through this procedure that the active path’s control performance will be enhanced if it is positioned in the optimal location and less control force is required than in the case of beam.

## Introduction

The main sources of noise, vibration, and harshness (NVH) in the fields of vehicle, aerospace, and mechanical engineering is the powertrain and they are transmitted to the whole structure through mounting systems. Furthermore, sensor and actuator placement in future mobility are crucial because when the positions and orientations are altered by excessive vehicle vibration, it results in the malfunctioning of autonomous driving systems^1–4^ To reduce or isolate them effectively, active attenuation methodologies using smart materials such as piezoelectric actuators are suggested due to their high precision and low weight^5–7^. A novel hybrid-mounted actuator consisting of an air spring and magneto-rheological dampers was proposed and acted on a heavy precision stage. The results show that the proposed hybrid actuator can significantly improve the vibration effect of the precision platform^8^. A control method for sensor and actuator is developed to apply to flexible beams and smart composite structures. Experimental results show that the proposed control method is feasible^9,10^. The Stewart platform can suppress vibration transmission with piezoelectric actuators based on adaptive feedback control of FxLMS. The results indicate that periodic and random disturbance are significantly attenuated in the low frequency range^11^. A solenoid actuator acting on the active engine mount is designed. The results show that the solenoid actuator can be applied to the active noise and vibration control system^12^. The combination of the Raspberry Pi 3 with sensors and actuators to attenuate vibration within the system link is proposed. Based on the Euler-Bernoulli beam theory, the analysis model of the structure was established, and the obtained modal parameters and frequency responses were verified through a finite element model and experimental data^13^. An active engine mount system with a hydraulic actuator is designed and controlled by an adaptive controller. The results show that the proposed method can effectively reduce vibration^14^. Passive and active components combined with a sliding mode controller show good control performance in a wide frequency range from 20 Hz to 1 kHz^15^. The performance of piezoelectric stack actuators in transmitting vibrations from the powertrain to the vehicle body has been demonstrated^16^. Piezoelectric actuators and a multiple NLMS algorithm are applied to active engine mounts, and the vibration reduction effect is very significant^17,18^. In addition, an active mounting system consisting of actuators and rubber mounts is used for active control. An adaptive controller for Active Engine Mount (AEM) is proposed according to the dynamic characteristics of hydraulic engine mount. Experiments have shown that the proposed system can reduce the force transmitted through the AEM^14^. A novel active mount using piezoelectric stack actuators and rubber elements is designed. The vibration control performance in time domain and frequency domain is verified^19^. A novel three-axis active mount using piezoelectric actuators is proposed and its effectiveness is verified through a vibration system subjected to vertical, rolling and pitching motions^20^ Vibration control is achieved by quantifying the interaction between two active structural paths for source mass motion. Simulations and experiments show that multi-level vibration isolation can be achieved^21^.

**Figure 1:**
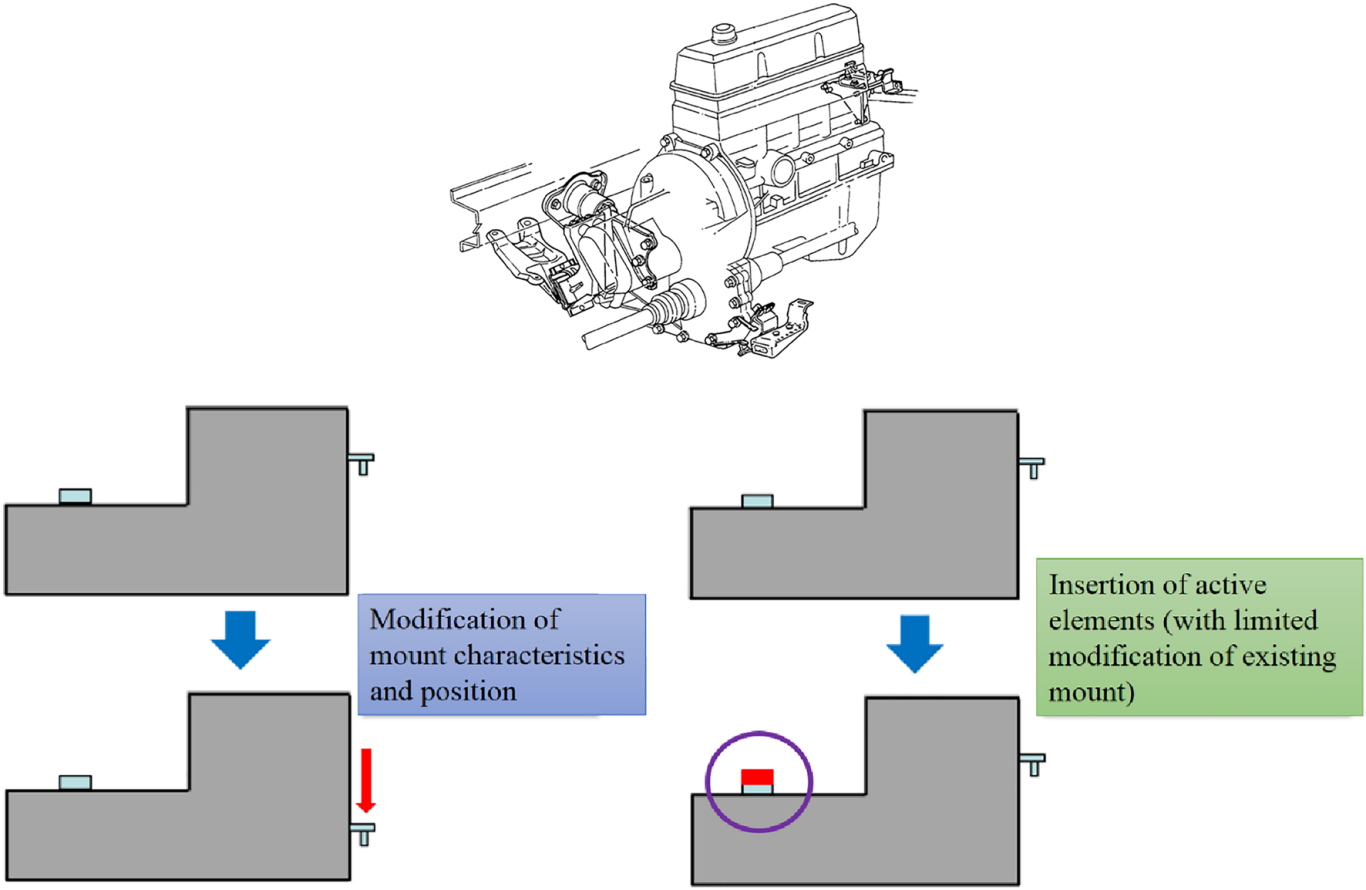
Two different ways of improving NVH performance of powertrain mounting system.

Figure 1 shows two different ways of improving NVH performance of powertrain mounting system. The left one describes an example to modify the position or characteristics (material, structure, etc.) of the mounts, which usually costs much and takes much time. The right one represents an example to just insert active elements with limited structural modification, whose time and cost is less than the previous case. In the literature review above, an active vibration control system based on an active mounting system has been demonstrated, but the optimal placement of piezoelectric actuators has not been proposed. The method of reducing vibration by controlling the actual engine is very expensive and difficult to commercialize. Furthermore, even with active mounting, optimal vibration control cannot be achieved because the location of the active mounting is usually designed without NVH performance in mind. Therefore, determining the optimal location of an active suspension system is critical to reducing control costs. Much research has focused on the optimal placement of active engine mounts to reduce vibrations.

Particularly, genetic algorithms^22^ and particle swarm optimization (PSO) algorithms^23,24^ are frequently used to fine-tune the optimal location. Based on the first-order shear deformation theory, a finite element model for optimal placement and active vibration control of laminated composite panels was developed. Using the optimal placement of piezoelectric patches, the stiffness of the structure can be increased^25^. An optimized algorithm is used to design the mounting system and the results show that the proposed method can reduce the vibration transmissibility^26^. The topological design of piezoelectric actuator layers is employed for the reduction of transient vibrations. It shows that the proposed method can enhance vibration control performance^27^. Through the study of optimization criteria for piezoelectric sensors and actuators, their locations are determined^28^. In addition, optimized placement of piezoelectric actuators using PSO could suppress excessive membrane structure vibration^29^. The optimal placement and geometry, determined by PSO, of piezoelectric sensors and actuators in laminated beam structures could improve control performance^30^. A novel method based on acoustic radiation is proposed to determine the optimal position of actuator^31^. A cost function with time-weighted controllability Gramian is proposed to determine the optimal position of the actuator. Test results show that optimally positioned actuators can damp a thin steel ring more effectively^32^.

The location optimization method proposed here uses simple static scheme to get mathematical explicit formula easily and the dynamic scheme is presented for analytical validation of the static scheme. Previous methods including GA, PSO and so on, cannot derive a generic expression for the optimized location but just obtain for a specific case given in each paper. In addition, the active path in those papers consisted solely of piezoelectric actuators. In this research, however, the active paths consist of an actuator and a rubber mount and it can be contrasted to the passive path to demonstrate the effect of active control by simply removing power from the active elements. In addition, the majority of previous works considered only a single active path, and the effect of multiple piezoelectric actuators operating in concert cannot be confirmed.

Analytical methods of dynamics and statics were used to determine the optimal location of the active engine mounting system applied to a 6-DOF beam structure, and the proposed criteria were experimentally validated^33^. However, the optimal position of the active mounting system applied to the 6-DOF beam can only be determined by considering the y-direction moment of inertia. Consequently, in this study, a non-aligned plate structure will be used to optimize the locations of vibration trajectories by taking the moments of inertia in both orientations into account.

### Problem formulation

**Figure 2:**
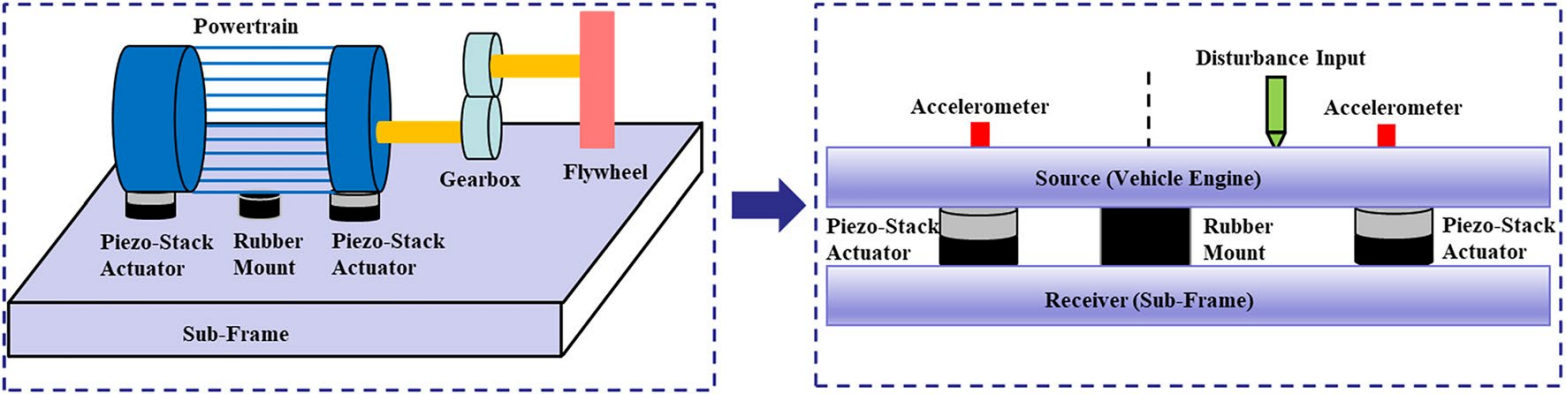
Active element integration for mitigating source movement from disturbance force.

The primary purpose of this paper is to propose optimal placement criteria for the active mounting system in a smart composite plate structure. To accomplish this, a smart composite plate structure is prepared depicted in Fig. 2. The upper plate represents the engine, while the lower plate represents the subframe. Additionally, the path segments include one passive path and two active paths. Passive paths consist of a rubber mount and a piezoelectric actuator, while active paths consist of a rubber mount and a piezoelectric actuator. These are the objectives of this paper: (1) Based on the lumped parameter model proposed, the control force formula for the two active paths is formulated. (2) A method for determining the optimal location of an active mounting system based on dynamic and static analysis is proposed. (3) A setup for a feasibility experiment is designed in order to validate the optimal location and control performance.

The given structure is assumed to be discrete, frequency independent, linear, and free of nonlinear kinematic effects around the operating point. Consequently, the superposition principle applies, the motion is relatively small, and the higher-order terms can be disregarded. In addition, it is assumed that each component is rigid, there is no bending, and the known external harmonic perturbation force excites only the source component. The control forces are derived analytically based on the steady-state behavior of the system. In the structural alignment, passive components are assumed to have no mass, whereas active components are assumed to have mass. Each spring element is assumed to have a constant damping coefficient for structural damping. The only motion of the structure is vertical, and the active elements are piezoelectric stack actuators.

## Mathematical modeling and control force

### Lumped parameter model

**Figure 3:**
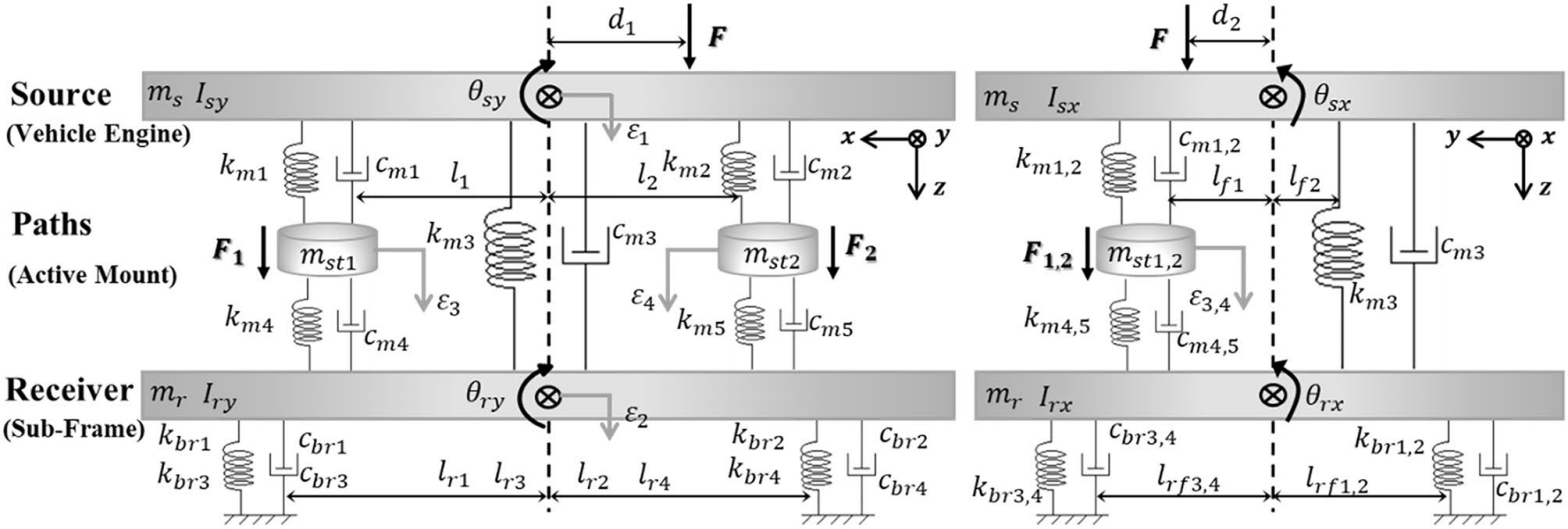
Modeling of given source-path-receiver system.

Fig. 3 depicts the suggested analytical model of the smart composite plate structure with concurrent active path and passive path based on the source-path-receiver system. Paths consist of an active path and a passive path, where source denotes a vehicle engine. For active vibration control, the active path consists of a piezoelectric actuator and a rubber mounting. The sub-frame is indicated by the receiver. The displacement is disregarded in the x and y-directions but is taken into account in the z-direction only. Also considered is rotational motion in the x and y-directions. Fig. 3 depicts the proposed analytical model, which consists of four translational motions and four rotational motions.

All the parameters are determined and used based on the experiment. ${m}_{s}$ and ${m}_{r}$ represent the mass corresponding to source and receiver, respectively. ${m}_{st1}$ and ${m}_{st2}$ represent the mass corresponding to actuator, respectively. ${\theta }_{sy}$ and ${\theta }_{ry}$ represent rotational motions in y-direction corresponding to source and receiver, respectively. ${I}_{sy}$ and ${I}_{ry}$ represent the inertia in y direction corresponding to the source and receiver, respectively. ${\theta }_{sx}$ and ${\theta }_{rx}$ represent the rotational motions in x-direction corresponding to source and receiver, respectively. ${I}_{sx}$ and ${I}_{rx}$ represent the inertia in x-direction corresponding to source and receiver, respectively. ${l}_{i}$ and ${l}_{ri}$ represent the lengths from center gravity to active mount and rubber mount. ${d}_{i}$ represents the lengths from center gravity to shaker. ${k}_{mi}, {k}_{bri}$ and ${c}_{mi}, {c}_{bri}$ represent the stiffness and damping coefficient.${F}_{1}\left(t\right), {F}_{2}(t)$ and $F(t)$ represent the force corresponding to actuator and the disturbance. ${\varepsilon }_{i}$ represents the vertical motions.

**Figure. 4:**
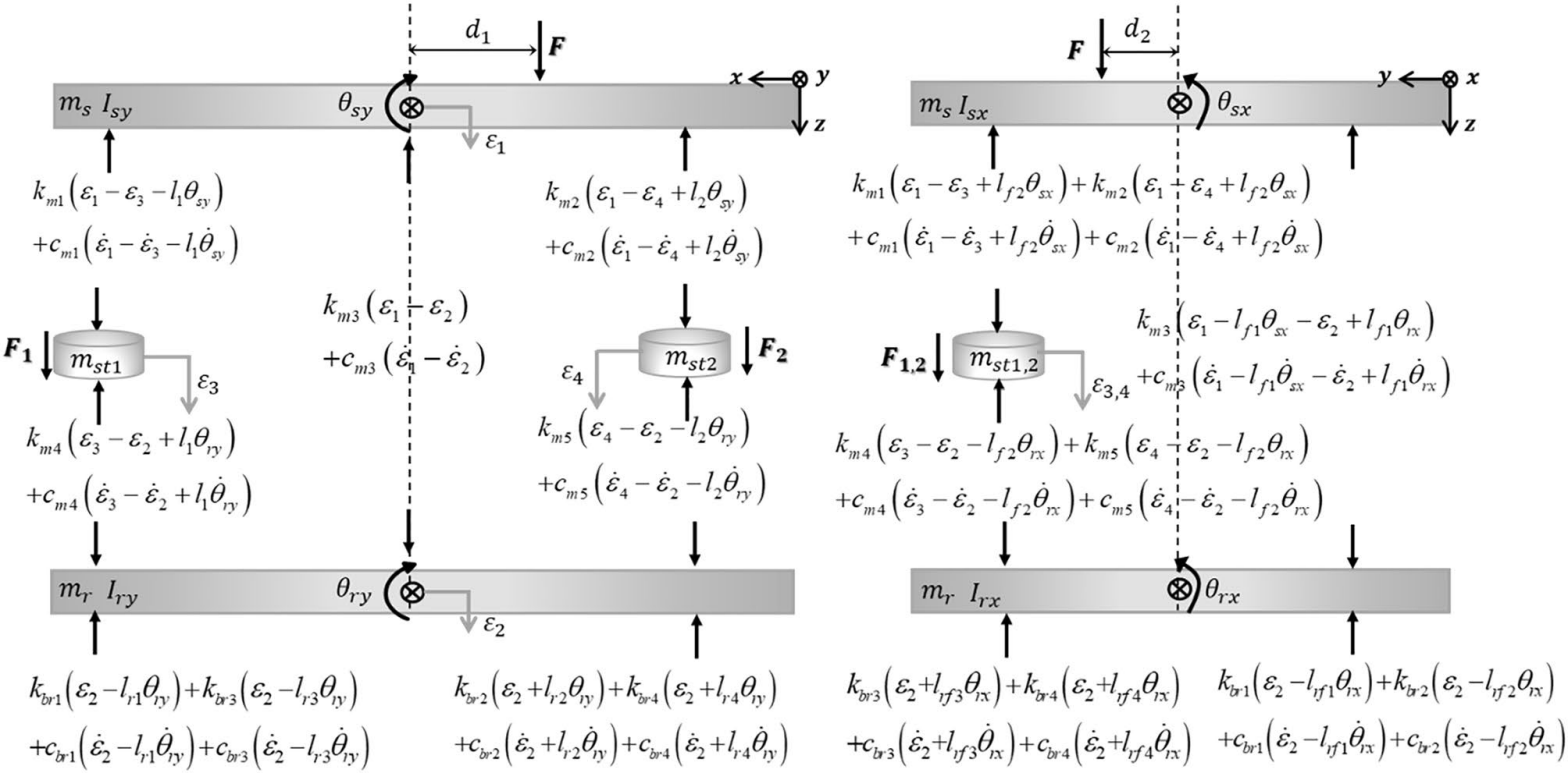
Free body diagram of system.

Free body diagram is presented in Fig. 4. Based on the Newton's 2^nd^ law, the equation of motion can be expressed by Eq. (1). Here, $M$, $K$, and $C$ represent mass, stiffness, and damping matrix, respectively. Also, $F(t)$, $q(t)$, and $W(t)$ represent control force, displacement, and disturbance force, respectively. The matrix of mass, stiffness, damping, required control force of actuator, displacement, and excitation force is summarized in Eq. (2)–(7). Also, the values of parameters are summarized in Table 1.

**Table 1:** Identified system parameters.

Parameter	Value	Unit
${m}_{s}={m}_{r}$	$2.14$	kg
${m}_{st1}={m}_{st2}$	$0.075$	kg
${I}_{sy}={I}_{ry}$	$19.44$	${\mathrm{gm}}^{2}$
${I}_{sx}={I}_{rx}$	$10.29$	${\mathrm{gm}}^{2}$
${k}_{m1}$	$5.64(1+\mathrm{i}0.036)$	${\mathrm{kN mm}}^{-1}$
${k}_{m2}$	$5.64(1+\mathrm{i}0.036)$	${\mathrm{kN mm}}^{-1}$
${k}_{m3}$	$0.61(1+\mathrm{i}0.034)$	${\mathrm{kN mm}}^{-1}$
${k}_{m4}$	$0.53(1+\mathrm{i}0.300)$	${\mathrm{kN mm}}^{-1}$
${k}_{m5}$	$0.53(1+\mathrm{i}0.300)$	${\mathrm{kN mm}}^{-1}$
${k}_{brn}$	$0.42(1+\mathrm{i}0.300)$	${\mathrm{kN mm}}^{-1}$
${d}_{1}$	$58$	$\mathrm{mm}$
${d}_{2}$	$10$	$\mathrm{mm}$
${l}_{2}$	$29, 101.5 \& 145$	$\mathrm{mm}$
${l}_{f1}$	$60$	$\mathrm{mm}$
${l}_{f2}$	60	$\mathrm{mm}$
${l}_{r1}={l}_{r2}={l}_{r3}={l}_{r4}$	$150$	$\mathrm{mm}$
${l}_{rf1}={l}_{rf2}={l}_{rf3}={l}_{rf4}$	$100$	$\mathrm{mm}$

1 $$M\ddot{q}\left(t\right)+C\dot{q}\left(t\right)+kq\left(t\right)=W\left(t\right)+F\left(t\right)$$

2 $$M=diag\left\{\begin{array}{cccccccc}{m}_{s}& {m}_{r}& {m}_{st1}& {m}_{st2}& {I}_{sy}& {I}_{ry}& {I}_{sx}& {I}_{rx}\end{array}\right\}$$

$$K=\left[\begin{array}{ccccc}{k}_{m1}+{k}_{m2}+{k}_{m3}& -{k}_{m3}& -{k}_{m1}& -{k}_{m2}& {k}_{m2}{l}_{2}-{k}_{m1}{l}_{1}\\ -{k}_{m3}& {k}_{m3}+{k}_{m4}+{k}_{m5}+{k}_{br1}+{k}_{br2}+{k}_{br3}+{k}_{br4}& -{k}_{m4}& -{k}_{m1}& 0\\ -{k}_{m1}& -{k}_{m4}& {k}_{m1}+{k}_{m4}& 0& {k}_{m1}{l}_{1}\\ -{k}_{m2}& -{k}_{m5}& 0& {k}_{m2}+{k}_{m5}& -{k}_{m2}{l}_{2}\\ {k}_{m2}{l}_{2}-{k}_{m1}{l}_{1}& 0& {k}_{m1}{l}_{1}& -{k}_{m2}{l}_{2}& {k}_{m1}{l}_{1}^{2}+{k}_{m2}{l}_{2}^{2}\\ 0& {k}_{m5}{l}_{2}-{k}_{m4}{l}_{1}-{k}_{br1}{l}_{r1}+{k}_{br2}{l}_{r2}-{k}_{br3}{l}_{r3}+{k}_{br4}{l}_{r4}& {k}_{m4}{l}_{1}& -{k}_{m5}{l}_{2}& 0\\ {k}_{m1}{l}_{f2}+{k}_{m2}{l}_{f2}-{k}_{m3}{l}_{f1}& {k}_{m3}{l}_{f1}& -{k}_{m1}{l}_{f2}& -{k}_{m2}{l}_{f2}& 0\\ {k}_{m3}{l}_{f1}& {k}_{m5}{l}_{f2}+{k}_{m4}{l}_{f2}-{k}_{m3}{l}_{f1}-{k}_{br1}{l}_{rf1}-{k}_{br2}{l}_{rf2}+{k}_{br3}{l}_{rf3}+{k}_{br4}{l}_{rf4}& -{k}_{m4}{l}_{f2}& -{k}_{m5}{l}_{f2}& 0\end{array}\right.$$

3 $$\left.\begin{array}{ccc}0& {k}_{m1}{l}_{f2}+{k}_{m2}{l}_{f2}-{k}_{m3}{l}_{f1}& {k}_{m3}{l}_{f1}\\ {-k}_{m4}{l}_{1}+{k}_{m5}{l}_{2}-{k}_{br1}{l}_{r1}+{k}_{br2}{l}_{r2}-{k}_{br3}{l}_{r3}+{k}_{br4}{l}_{r4}& {k}_{m3}{l}_{f1}& {k}_{m4}{l}_{f2}+{k}_{m5}{l}_{f1}-{k}_{m3}{l}_{f1}-{k}_{br1}{l}_{rf1}-{k}_{br2}{l}_{rf2}+{k}_{br3}{l}_{rf3}+{k}_{br4}{l}_{rf4}\\ {k}_{m4}{l}_{1}& -{k}_{m1}{l}_{f2}& -{k}_{m4}{l}_{f2}\\ -{k}_{m5}{l}_{2}& -{k}_{m2}{l}_{f2}& -{k}_{m5}{l}_{f2}\\ 0& 0& 0\\ {k}_{m4}{l}_{1}^{2}+{k}_{m5}{l}_{2}^{2}+{k}_{br1}{l}_{r1}^{2}+{k}_{br2}{l}_{r2}^{2}+{k}_{br3}{l}_{r3}^{2}+{k}_{br4}{l}_{r4}^{2}& 0& 0\\ 0& {k}_{m3}{l}_{f1}^{2}+{k}_{m1}{l}_{f2}^{2}+{k}_{m2}{l}_{f2}^{2}& -{k}_{m3}{l}_{f1}^{2}\\ 0& -{k}_{m3}{l}_{f1}^{2}& {k}_{m4}{l}_{f2}^{2}+{k}_{m5}{l}_{f2}^{2}+{k}_{m3}{l}_{f1}^{2}+{k}_{br1}{l}_{rf1}^{2}+{k}_{br2}{l}_{rf2}^{2}+{k}_{br3}{l}_{rf3}^{2}+{k}_{br4}{l}_{br4}^{2}\end{array}\right]$$

$$C=\left[\begin{array}{cccc}{c}_{m1}+{c}_{m2}+{c}_{m3}& -{c}_{m3}& -{c}_{m1}& -{c}_{m2}\\ -{c}_{m3}& {c}_{m3}+{c}_{m4}+{kc}_{m5}+{c}_{br1}+{c}_{br2}+{c}_{br3}+{c}_{br4}& -{c}_{m4}& -{c}_{m1}\\ -{c}_{m1}& -{c}_{m4}& {c}_{m1}+{c}_{m4}& 0\\ -{c}_{m2}& -{c}_{m5}& 0& {c}_{m2}+{c}_{m5}\\ {c}_{m2}{l}_{2}-{c}_{m1}{l}_{1}& 0& {c}_{m1}{l}_{1}& -{c}_{m2}{l}_{2}\\ 0& {c}_{m5}{l}_{2}-{c}_{m4}{l}_{1}-{c}_{br1}{l}_{r1}+{c}_{br2}{l}_{r2}-{c}_{br3}{l}_{r3}+{c}_{br4}{l}_{r4}& {c}_{m4}{l}_{1}& -{c}_{m5}{l}_{2}\\ {c}_{m1}{l}_{f2}+{c}_{m2}{l}_{f2}-{c}_{m3}{l}_{f1}& {c}_{m3}{l}_{f1}& -{c}_{m1}{l}_{f2}& -{c}_{m2}{l}_{f2}\\ {c}_{m3}{l}_{f1}& {c}_{m5}{l}_{f2}+{c}_{m4}{l}_{f2}-{c}_{m3}{l}_{f1}-{c}_{br1}{l}_{rf1}-{c}_{br2}{l}_{rf2}+{c}_{br3}{l}_{rf3}+{c}_{br4}{l}_{rf4}& -{c}_{m4}{l}_{f2}& -{c}_{m5}{l}_{f2}\end{array}\right.$$

4 $$\left.\begin{array}{cccc}{c}_{m2}{l}_{2}-{c}_{m1}{l}_{1}& 0& {c}_{m1}{l}_{f2}+{c}_{m2}{l}_{f2}-{c}_{m3}{l}_{f1}& {c}_{m3}{l}_{f1}\\ 0& {-c}_{m4}{l}_{1}+{c}_{m5}{l}_{2}-{c}_{br1}{l}_{r1}+{c}_{br2}{l}_{r2}-{c}_{br3}{l}_{r3}+{c}_{br4}{l}_{r4}& {c}_{m3}{l}_{f1}& {c}_{m4}{l}_{f2}+{c}_{m5}{l}_{f1}-{c}_{m3}{l}_{f1}-{c}_{br1}{l}_{rf1}-{c}_{br2}{l}_{rf2}+{c}_{br3}{l}_{rf3}+{c}_{br4}{l}_{rf4}\\ {c}_{m1}{l}_{1}& {c}_{m4}{l}_{1}& -{c}_{m1}{l}_{f2}& -{c}_{m4}{l}_{f2}\\ -{c}_{m2}{l}_{2}& -{c}_{m5}{l}_{2}& -{c}_{m2}{l}_{f2}& -{c}_{m5}{l}_{f2}\\ {c}_{m1}{l}_{1}^{2}+{c}_{m2}{l}_{2}^{2}& 0& 0& 0\\ 0& {c}_{m4}{l}_{1}^{2}+{c}_{m5}{l}_{2}^{2}+{c}_{br1}{l}_{r1}^{2}+{c}_{br2}{l}_{r2}^{2}+{c}_{br3}{l}_{r3}^{2}+{c}_{br4}{l}_{r4}^{2}& 0& 0\\ 0& 0& {c}_{m3}{l}_{f1}^{2}+{c}_{m1}{l}_{f2}^{2}+{c}_{m2}{l}_{f2}^{2}& -{c}_{m3}{l}_{f1}^{2}\\ 0& 0& -{c}_{m3}{l}_{f1}^{2}& {c}_{m4}{l}_{f2}^{2}+{c}_{m5}{l}_{f2}^{2}+{c}_{m3}{l}_{f1}^{2}+{c}_{br1}{l}_{rf1}^{2}+{c}_{br2}{l}_{rf2}^{2}+{c}_{br3}{l}_{rf3}^{2}+{c}_{br4}{l}_{br4}^{2}\end{array}\right]$$

5 $$F\left(t\right)={\left[\begin{array}{cccccccc}0& 0& {F}_{1}(t)& {F}_{2}(t)& 0& 0& 0& 0\end{array}\right]}^{T}$$

6 $$q\left(t\right)={\left[\begin{array}{cccccccc}{\varepsilon }_{1}(t)& {\varepsilon }_{2}(t)& {\varepsilon }_{3}(t)& {\varepsilon }_{4}(t)& {\theta }_{sy}(t)& {\theta }_{ry}(t)& {\theta }_{sx}(t)& {\theta }_{rx}(t)\end{array}\right]}^{T}$$

7 $$W\left(t\right)={\left[\begin{array}{cccccccc}F\left(t\right)& 0& 0& 0& F\left(t\right){d}_{1}& 0& F\left(t\right){d}_{2}& 0\end{array}\right]}^{T}$$

The parameter values of system are shown in Table 1 and they are from the experimental setup presented in this article in the later section. The masses of plates (source and receiver) are weighed, lengths are measured, and moments of inertia are calculated. Stiffness values of actuator and rubbers are represented as complex values with the following expressions, $k_{{mi}} = k{^{{ \wedge }}} _{{mi}} \left( {1 + i\eta _{{mi}} } \right)$ and $k_{{bri}} = k{^{{ \wedge }}}_{{brii}} (\left( {1 + i\eta _{{bri}} } \right)\eta _{{mi}}$ ,where $k{^{{ \wedge }}} _{{mi}}$ and $k{^{{ \wedge }}}_{{brii}}$ represent the real part, $\eta_{mi}$ and $\eta_{bri}$ represent the loss factor. These values are determined with simple experiments as follows: (1) a chirp voltage signal acted on the actuator, (2) the resonant frequency of the actuator is obtained, (3) the stiffness values are calculated with the following formulation for example, $k = \omega^{2} m_{st1}$, where $\omega$ is the natural frequency, (4) the loss factors are calculated using half-power method with the formulation of $\eta = \left( {\omega_{2} - \omega_{1} } \right)/\omega$, where $\omega_{1}$ and $\omega_{2}$ are the half-power frequencies.

The equation of motion is derived for the coordinates of the center of mass. To confirm the vibration-reduction effect of active mounting systems, it is necessary to relocate the center of mass coordination to the location of each active mounting system.

**Figure 5:**
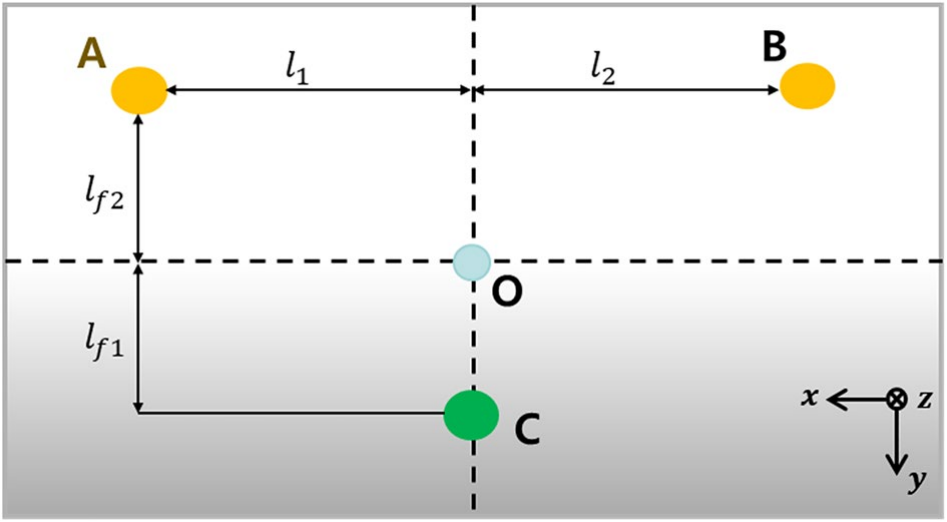
Positional relationship between actuator 1, actuator 2 and rubber mount (Top view of the system).

Fig. 5 depicts the relationship between the center of mass and active mounts, and Eq. (8) defines the corresponding mathematical. In Fig. 5, *A*, *B*, *C*, and *O* represent, respectively, the positions corresponding to actuator 1 (path1), actuator 2 (path2), rubber mount (path3), and the center of mass.

8 $$O=\frac{{l}_{f2}}{{l}_{f1}+{l}_{f2}}C+\left(\frac{{l}_{f1}}{{l}_{f1}+{l}_{f2}}\frac{{l}_{2}}{{l}_{1}+{l}_{2}}\right)A+\left(\frac{{l}_{f1}}{{l}_{f1}+{l}_{f2}}\frac{{l}_{1}}{{l}_{1}+{l}_{2}}\right)B$$

Using Eq. (8), the transfer matrix $\Pi$ can be defined as in Eq. (9).

9 $$\Pi =\left[\begin{array}{cccccccc}\frac{{l}_{f1}\cdot {l}_{2}}{\left({l}_{f1}+{l}_{f2}\right)\left({l}_{1}+{l}_{2}\right)}& \frac{{l}_{f1}\cdot {l}_{1}}{\left({{l}_{f1}+l}_{f2}\right)\left({l}_{1}+{l}_{2}\right)}& \frac{{l}_{f2}}{{l}_{f1}+{l}_{f2}}& 0& 0& 0& 0& 0\\ 0& 0& 0& 0& 0& \frac{{l}_{f1}\cdot {l}_{2}}{\left({l}_{f1}+{l}_{f2}\right)\left({l}_{1}+{l}_{2}\right)}& \frac{{l}_{f1}\cdot {l}_{1}}{\left({{l}_{f1}+l}_{f2}\right)\left({l}_{1}+{l}_{2}\right)}& \frac{{l}_{f2}}{{l}_{f1}+{l}_{f2}}\\ 0& 0& 0& 1& 0& 0& 0& 0\\ 0& 0& 0& 0& 1& 0& 0& 0\\ -\frac{1}{\left({l}_{f1}+{l}_{f2}\right)\left({l}_{1}+{l}_{2}\right)}& \frac{1}{\left({l}_{f1}+{l}_{f2}\right)\left({l}_{1}+{l}_{2}\right)}& 0& 0& 0& 0& 0& 0\\ 0& 0& 0& 0& 0& -\frac{1}{\left({l}_{f1}+{l}_{f2}\right)\left({l}_{1}+{l}_{2}\right)}& \frac{1}{\left({l}_{f1}+{l}_{f2}\right)\left({l}_{1}+{l}_{2}\right)}& 0\\ \begin{array}{c}\frac{1}{\left({l}_{f1}+{l}_{f2}\right)\left({l}_{1}+{l}_{2}\right)}\\ 0\end{array}& \frac{1}{\left({l}_{f1}+{l}_{f2}\right)\left({l}_{1}+{l}_{2}\right)}& -\frac{1}{{l}_{f1}+{l}_{f2}}& 0& 0& 0& 0& 0\\ 0& 0& 0& 0& 0& \begin{array}{c}0\\ \frac{1}{\left({l}_{f1}+{l}_{f2}\right)\left({l}_{1}+{l}_{2}\right)}\end{array}& \frac{1}{\left({l}_{f1}+{l}_{f2}\right)\left({l}_{1}+{l}_{2}\right)}& -\frac{1}{{l}_{f1}+{l}_{f2}}\end{array}\right]$$

**Figure 6:**
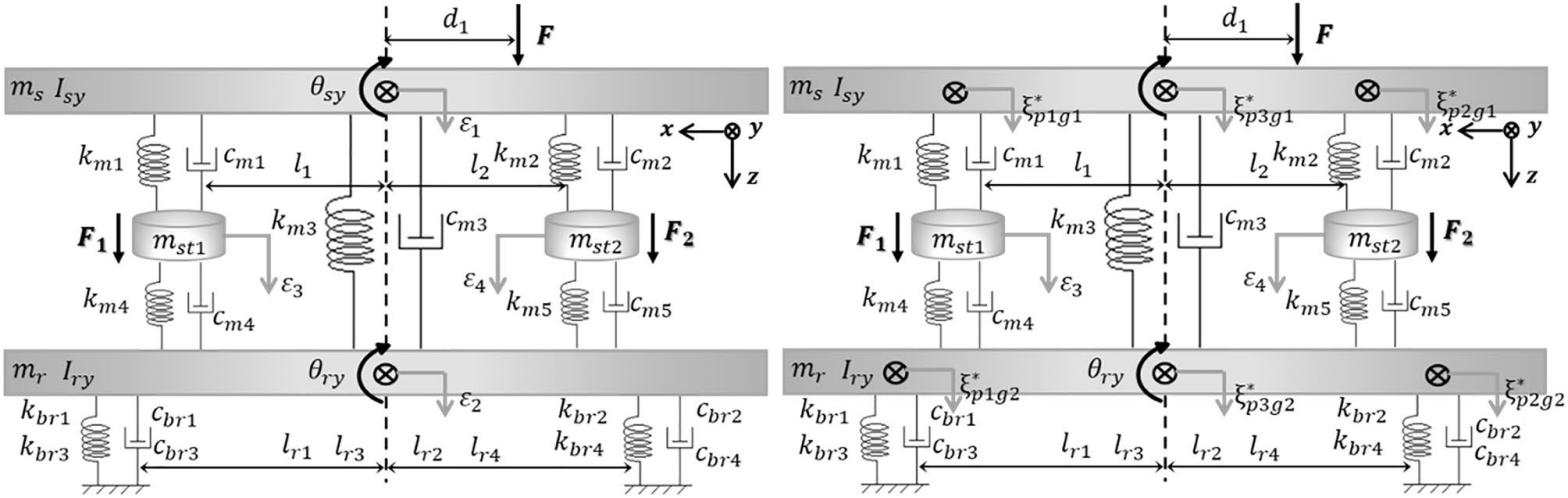
Schematic representation: (**a**) Mass coordinates and (**b**) Mount coordinates.

The coordinate transformation is performed based on Eq. (9), and the transformed coordinate is depicted in Fig. 6. The expressions for the transformed displacement, matrix, and stiffness are as shown in Eq. (10). According to the following definition, the new equation of motion is defined as follows Eq. (11). The displacement vector corresponding to each path is defined as Eq. (12).

10 $$q(t)=\Pi {q}^{*}(t) {M}^{*}=M\Pi {K}^{*}=K\Pi$$

11 $${M}^{*}{\ddot{q}}^{*}\left(t\right)+{C}^{*}{\dot{q}}^{*}(t)+{K}^{*}{q}^{*}(t)=F\left(t\right)+W(t)$$

12 $${q}^{*}\left(t\right)={\left\{\begin{array}{cccccccc}{\xi }_{p1g1}^{*}& {\xi }_{p2g1}^{*}& {\xi }_{p3g1}^{*}& {\varepsilon }_{3}& {\varepsilon }_{4}& {\xi }_{p1g2}^{*}& {\xi }_{p2g2}^{*}& {\xi }_{p3g2}^{*}\end{array}\right\}}^{T}$$

### Control force derivation

When considering the system motion, the phase between harmonic force and motion play an important role. Thus, the disturbance and control forces are assumed to be complex valued. It is defined as follows Eq. (13) to Eq. (14).

13 $${F}^{*}\left(t\right)={W}_{1}{e}^{i\omega t}$$

14 $${F}_{i}^{*}\left(t\right)={F}_{i}{e}^{i\left(\omega t+{\mathrm{\varnothing }}_{sti}\right)}$$

where ${W}_{1}$ represents the amplitude corresponding to the shaker. ${F}_{i}$ represent the amplitude corresponding to the *ith* actuator. ${\phi }_{sti}$ represent the phase corresponding to *ith* actuator. In order to simplify the notation, the displacement of each active path is redefined as following Eq. (15).

15 $${\xi }_{i}^{*}\left(t\right)={\xi }_{pig1}^{*}(t)$$

In order to isolate the vibration, ${\xi }_{i}^{*}(t)$ should be minimized. Since the system is linear, therefore the source mass movement can be defined as following Eq. (16).

16 $${\xi }_{i}^{*}\left(t\right)=\left({\Xi }_{si,1}^{*}+{\Xi }_{si,3}^{*}{e}^{i{\varnothing }_{st1}}+{\Xi }_{si,4}^{*}{e}^{i{\varnothing }_{st2}}\right){e}^{i\omega t}$$

In Eq. (16), ${\Xi }_{si,1}^{*}$ represent the complex valued amplitude of the *i*th path due to ${w}_{1}^{*}(t)$, ${\Xi }_{si,3}^{*}$ and ${\Xi }_{si,4}^{*}$ represent the complex valued amplitudes due to actuator forces. The dynamic stiffness matrix is defined as $\widetilde{\kappa }={\left[-{\omega }^{2}{M}{\prime}+{K}{\prime}\right]}^{-1}$. According to formula ${H}^{*{\prime}}={\left[{\kappa }{\prime}\right]}^{-1}$, compliance matrix can be obtained as Eq. (17).

17 $${H}^{*{\prime}}=\left[\begin{array}{cccccccc}{H}_{11}^{*{\prime}}& {H}_{12}^{*{\prime}}& {H}_{13}^{*{\prime}}& {H}_{14}^{*{\prime}}& {H}_{15}^{*{\prime}}& {H}_{16}^{*{\prime}}& {H}_{17}^{*{\prime}}& {H}_{18}^{*{\prime}}\\ {H}_{21}^{*{\prime}}& {H}_{22}^{*{\prime}}& {H}_{23}^{*{\prime}}& {H}_{24}^{*{\prime}}& {H}_{25}^{*{\prime}}& {H}_{26}^{*{\prime}}& {H}_{27}^{*{\prime}}& {H}_{28}^{*{\prime}}\\ {H}_{31}^{*{\prime}}& {H}_{32}^{*{\prime}}& {H}_{33}^{*{\prime}}& {H}_{34}^{*{\prime}}& {H}_{35}^{*{\prime}}& {H}_{36}^{*{\prime}}& {H}_{37}^{*{\prime}}& {H}_{38}^{*{\prime}}\\ {H}_{41}^{*{\prime}}& {H}_{42}^{*{\prime}}& {H}_{43}^{*{\prime}}& {H}_{44}^{*{\prime}}& {H}_{45}^{*{\prime}}& {H}_{46}^{*{\prime}}& {H}_{47}^{*{\prime}}& {H}_{48}^{*{\prime}}\\ {H}_{51}^{*{\prime}}& {H}_{52}^{*{\prime}}& {H}_{53}^{*{\prime}}& {H}_{54}^{*{\prime}}& {H}_{55}^{*{\prime}}& {H}_{56}^{*{\prime}}& {H}_{57}^{*{\prime}}& {H}_{58}^{*{\prime}}\\ {H}_{61}^{*{\prime}}& {H}_{62}^{*{\prime}}& {H}_{63}^{*{\prime}}& {H}_{64}^{*{\prime}}& {H}_{65}^{*{\prime}}& {H}_{66}^{*{\prime}}& {H}_{67}^{*{\prime}}& {H}_{68}^{*{\prime}}\\ {H}_{71}^{*{\prime}}& {H}_{72}^{*{\prime}}& {H}_{73}^{*{\prime}}& {H}_{74}^{*{\prime}}& {H}_{75}^{*{\prime}}& {H}_{76}^{*{\prime}}& {H}_{77}^{*{\prime}}& {H}_{78}^{*{\prime}}\\ {H}_{81}^{*{\prime}}& {H}_{82}^{*{\prime}}& {H}_{83}^{*{\prime}}& {H}_{84}^{*{\prime}}& {H}_{85}^{*{\prime}}& {H}_{86}^{*{\prime}}& {H}_{87}^{*{\prime}}& {H}_{88}^{*{\prime}}\end{array}\right]$$

In order to calculate the amplitude and phase at the mount coordinates, the compliance matrix is utilized to determine the amount of deformation caused by the unit load. For the purpose of calculating the amplitude at each mounting point, the amplitude corresponding to each position is defined as follows Eq. (18).

18 $${Q}^{*{\prime}}=\left\{\begin{array}{cccccccc}{\Xi }_{p1g1}^{*}& {\Xi }_{p2g1}^{*}& {\Xi }_{p3g1}^{*}& {\zeta }_{3}& {\zeta }_{4}& {\Xi }_{p1g2}^{*}& {\Xi }_{p2g2}^{*}& {\Xi }_{p3g2}^{*}\end{array}\right\}$$

Using Eq. (17), the complex valued amplitude and phase due to the disturbance and actuator force are defined as Eqs. (19) and (20).

19 $${\Xi }_{si,1}^{*}=\left({H}_{i1}^{*{\prime}}+{H}_{i5}^{*{\prime}}{d}_{1}+{H}_{i7}^{*{\prime}}{d}_{2}\right){W}_{1}, {\Xi }_{si,3}^{*}={H}_{i3}^{*{\prime}}{F}_{i}, {\Xi }_{si,4}^{*}={H}_{i4}^{*{\prime}}{F}_{i}$$

20 $${\beta }_{si,1}=\angle \left({H}_{i1}^{*{\prime}}+{H}_{i5}^{*{\prime}}{d}_{1}+{H}_{i7}^{*{\prime}}{d}_{2}\right),{\beta }_{si,3}=\angle {H}_{i3}^{*{\prime}},{\beta }_{si,4}=\angle {H}_{i4}^{*{\prime}}$$

In Eq. (20), ${\beta }_{si,1}$ stands for the phase generated by the disturbance force on the *i*th path. $\angle$ represent the phase operator. Equation (16) is rewritten in terms of magnitude and phase as shown in the following formula Eq. (21).

21 $${\xi }_{i}^{*}\left(t\right)=\left(\left|{\Xi }_{si,1}^{*}\right|{e}^{i{\beta }_{si,1}}+\left|{\Xi }_{si,3}^{*}\right|{e}^{i{(\beta }_{si,3}+{\varnothing }_{st1})}+\left|{\Xi }_{si,4}^{*}\right|{e}^{i{(\beta }_{si,4}+{\varnothing }_{st2})}\right){e}^{i\omega t}$$

It complicates the motion control in Eq. (21) because it contains a multiphase term. Thus, in order to perform the control, it is necessary to perform the phase match. To achieve this, the phase match to disturbance force is used. This assumption allows the out-of-phase motion to be generated. So, *ith* actuator phase term ${\phi }_{sti}$ is defined as in Eq. (22) to perform the phase match.

22 $${\varnothing }_{sti}={\beta }_{si,1}-{\beta }_{si,\mathrm{3,4}}$$

Substituting the Eq. (22) into Eq. (21), so the movements of each path are rewritten as shown in Eq. (23).

23 $${\xi }_{i}^{*}\left(t\right)=\left(\left|{\Xi }_{si,1}^{*}\right|+\left|{\Xi }_{si,3}^{*}\right|+\left|{\Xi }_{si,4}^{*}\right|\right){e}^{i(\omega t+{\beta }_{si,1})}$$

In order to reduce the motion to zero, the magnitude part is assumed to zero, and it can be written as Eq. (24) and Eq. (25).

24 $$\left|{\Xi }_{s\mathrm{1,1}}^{*}\right|+\left|{\Xi }_{s\mathrm{1,3}}^{*}\right|+\left|{\Xi }_{s\mathrm{1,4}}^{*}\right|=0\Rightarrow {W}_{1}\left|{H}_{11}^{*}+{H}_{15}^{*}{d}_{1}+{H}_{17}^{*}{d}_{2}\right|+{F}_{1}\left|{H}_{13}^{*}\right|+{F}_{2}\left|{H}_{14}^{*}\right|=0$$

25 $$\left|{\Xi }_{s\mathrm{2,1}}^{*}\right|+\left|{\Xi }_{s\mathrm{2,3}}^{*}\right|+\left|{\Xi }_{s\mathrm{2,4}}^{*}\right|=0\Rightarrow {W}_{1}\left|{H}_{21}^{*}+{H}_{25}^{*}{d}_{1}+{H}_{27}^{*}{d}_{2}\right|+{F}_{1}\left|{H}_{23}^{*}\right|+{F}_{2}\left|{H}_{24}^{*}\right|=0$$

Through that assumption, the source motion can be reduced to zero. Using the Eqs. (24) and (25), the control force ${F}_{1}$ and ${F}_{2}$ can be defined as Eq. (26).

26 $$\begin{gathered} F_{1} = W_{1} \left( {\frac{{\left| {H_{14}^{*^{\prime}} } \right|\left| {H_{21}^{*^{\prime}} + H_{25}^{*^{\prime}} d_{1} + H_{27}^{*^{\prime}} d_{2} } \right| - \left| {H_{24}^{*^{\prime}} } \right|\left| {H_{11}^{*^{\prime}} + H_{15}^{*^{\prime}} d_{1} + H_{17}^{*^{\prime}} d_{2} } \right|}}{{\left| {H_{13}^{*^{\prime}} } \right|\left| {H_{24}^{*^{\prime}} } \right| - \left| {H_{14}^{*^{\prime}} } \right|\left| {H_{23}^{*^{\prime}} } \right|}}} \right) \hfill \\ F_{2} = W_{1} \left( {\frac{{\left| {H_{23}^{*^{\prime}} } \right|\left| {H_{11}^{*^{\prime}} + H_{15}^{*^{\prime}} d_{1} + H_{17}^{*^{\prime}} d_{2} } \right| - \left| {H_{13}^{*^{\prime}} } \right|\left| {H_{21}^{*^{\prime}} + H_{25}^{*^{\prime}} d_{1} + H_{27}^{*^{\prime}} d_{2} } \right|}}{{\left| {H_{13}^{*^{\prime}} } \right|\left| {H_{24}^{*^{\prime}} } \right| - \left| {H_{14}^{*^{\prime}} } \right|\left| {H_{23}^{*^{\prime}} } \right|}}} \right) \hfill \\ \end{gathered}$$

### Optimal placement of active mounting system

This section proposes a mathematical analysis for determining the optimal placement of an active mounting system using dynamic and static schemes. In conclusion, the objectives of this section are as follows: the optimal placement is determined after calculating the control force on each path using Eq. (26). Second, to validate the dynamic analysis, a mathematical formula based on the static method is proposed.

#### Dynamic scheme

**Figure 7:**
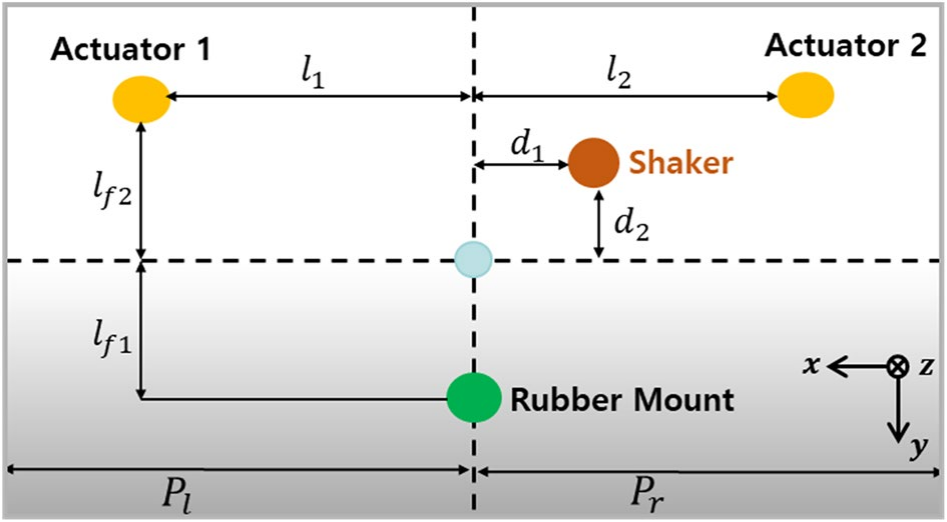
Relationship between shaker, actuators, and rubber mount (Top view of the system).

A schematic of the layout of actuator, shaker and rubber mount is shown in Fig. 7. The actuator 2 and shaker are placed at the right side of the plate structure. In Fig. 7, ${l}_{2}$ represents the length between center of gravity and actuator 2 in x-direction. Also, ${d}_{1}$ and ${d}_{2}$ represent the length between the center of gravity and shaker in x and y-direction, respectively. The actuator 1 is placed in the left side of the plate. ${l}_{1}$ represents the length between the center of gravity and actuator 1 in x-direction. ${l}_{f1}$ and ${l}_{f2}$ denotes the length of rubber mount and actuator in y-direction, respectively. ${P}_{l}$ and ${P}_{r}$ represent the length of plate and it is limited to 160 mm.

**Figure 8:**
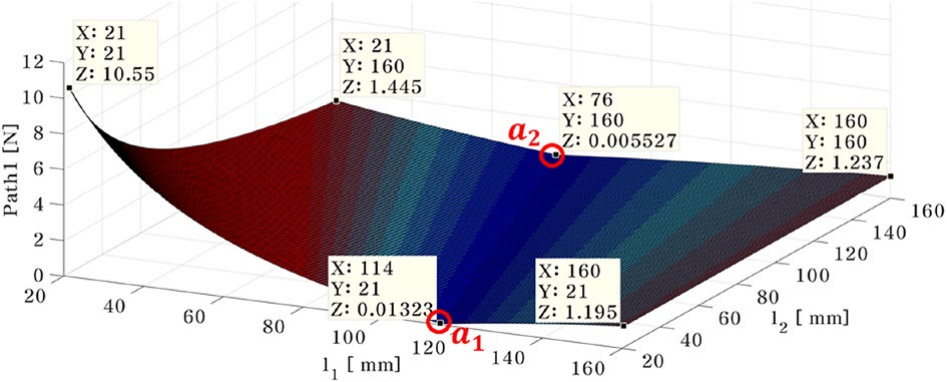
Needed control force on path 1—shaker placed in 58mm ($${d}_{1})$$ and 10mm ($${d}_{2})$$ (*generated with MATLAB R2016b*).

**Figure 9:**
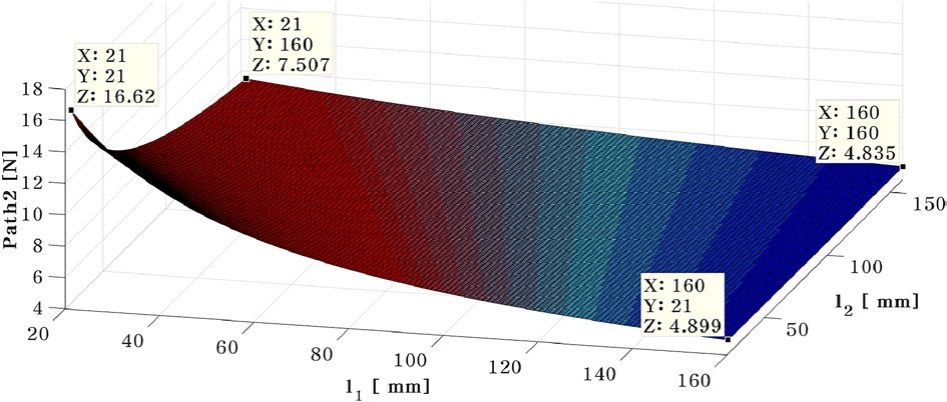
Needed control force on path 2 – shaker placed in 58mm ($${d}_{1})$$ and 10mm ($${d}_{2})$$ (*generated with MATLAB R2016b*).

To perform the optimal position analysis when the active mounting system and shaker are placed at different locations, the required control force of the actuator is computed using Eq. (26). Here, actuators 1 and 2 move from the plate's vicinity of the plate's center to its end edges. These lengths are limited to $29\le {l}_{1}\le {P}_{l}$ and $29\le {l}_{2}\le {P}_{r}$ at that time. Changes are made to the shaker's fixed 58 mm location. When the shaker is positioned at 58 mm and 10 mm in the x and y directions, respectively, Figs. 8 and 9 depict the results of the required control force on paths 1 and 2.

In Fig. 8, the simulation results show that regardless of the position of actuator 2, the required control force on the path 1 decreases. In addition, a greater control force is required when actuator 1 is positioned near the plate’s center. When the actuator is positioned in the blue range, from ${a}_{1}=(114, 21, 0.0132)$ to ${a}_{2}=(76, 160, 0.0055)$, the control force is minimized.

**Figure 10:**
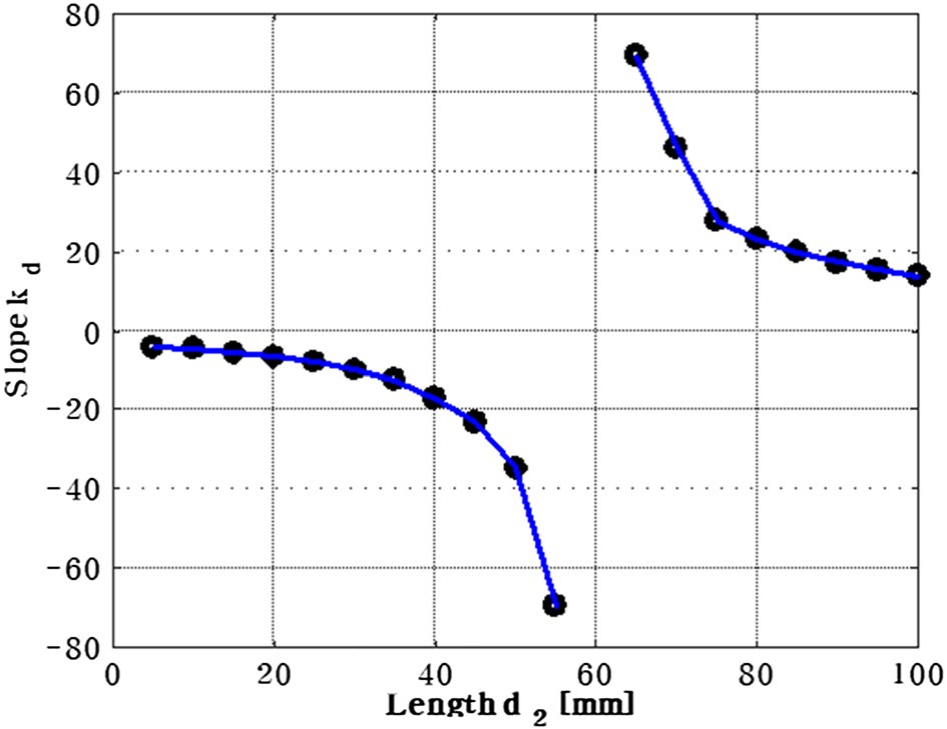
Relationship between the ${k}_{d}$ and ${d}_{2}$ (*generated with MATLAB R2016b*).

The required control force on path 2 is depicted in Fig. 9. When the position of actuator 1 is moved from near the plate's center to its edge, the required force decreases. Moreover, when comparing the forces of actuators 1 and 2, actuator 2 has a greater force than actuator 1 because it is positioned in the same location as the shaker. In order to confirm the effect of shaker location, the position of the shaker was varied by 5 mm, and the results are summarized in Table 2 and Fig. 10. In Table 2, ${a}_{1}$ and ${a}_{2}$ represent the range of actuator location corresponding to minimized actuator force. Additionally, the slope ${k}_{d}$ between point ${a}_{1}$ and ${a}_{2}$ is expressed as shown in Eq. (27).

**Table 2:** The placement of actuator 1, actuator 2 when the control force of actuator 1 is minimized, according to change the position of shaker from 5 to 100 mm.

${d}_{2}$(mm)	${a}_{1}({x}_{1},{y}_{1},{z}_{1})$	${a}_{2}({x}_{2},{y}_{2},{z}_{2})$	${k}_{d}(\mathrm{slope})$
5	(114, 21, 0.01320)	(76, 160, 0.00553)	$-3.971$
10	(105, 21, 0.01590)	(73, 160, 0.00008)	$-4.343$
15	(96, 21, 0.02237)	(70, 160, 0.00811)	$-5.346$
20	(90, 21, 0.02537)	(68, 160, 0.00091)	$-6.318$
25	(84, 21, 0.02521)	(66, 160, 0.00184)	$-7.722$
30	(78, 21, 0.03154)	(64, 160, 0.01110)	$-9.928$
35	(74, 21, 0.00643)	(63, 160, 0.00498)	$-12.636$
40	(70, 21, 0.00671)	(62, 160, 0.01700)	$-17.375$
45	(66, 21, 0.03589)	(60, 160, 0.01345)	$-23.166$
50	(63, 21, 0.01949)	(59, 160, 0.01199)	$-34.750$
55	(60, 21, 0.03644)	(58, 160, 0.01479)	$-69.500$
60	(58, 21, 0.03565)	(58, 160, 0.02341)	$0$
65	(55, 21, 0.04773)	(57, 160, 0.01432)	69.500
70	(53, 21, 0.02780)	(56, 160, 0.00082)	46.333
75	(51, 21, 0.03267)	(56, 160, 0.01713)	27.800
80	(49, 21, 0.06446)	(55, 160, 0.01510)	23.166
85	(48, 21, 0.05322)	(55, 160, 0.04730)	19.857
90	(46, 21, 0.02614)	(54, 160, 0.00208)	17.375
95	(44, 21, 0.06214)	(53, 160, 0.01037)	15.444
100	(43, 21, 0.07163)	(53, 160, 0.01779)	13.900

27 $${k}_{d}={y}_{2}-{y}_{1}/{x}_{2}-{x}_{1}$$

When ${d}_{2}<60mm$, the slope ${k}_{d}$ is significantly increased and when ${d}_{2}>60mm$, the slope ${k}_{d}$ is reduced. If the shaker is located in ${d}_{2}=60mm$, i.e., the ${k}_{d}$ has a zero, at that time, the shaker, the actuator 1, and actuator 2 are located in a straight line (aligned). It means that the optimized location of actuator 1 can be placed to the opposite of disturbance force even actuator 2 is located in any position.

#### Static scheme

**Figure 11:**
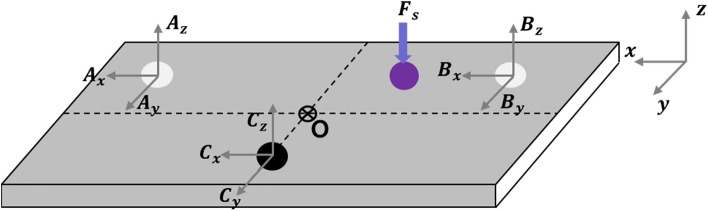
Statics analysis diagram for three paths.

In this section, the optimal placement criteria for the active mounting system are determined using a static analysis. The equilibrium equation of force and moment on each path is determined, and Fig. 11 depicts a schematic of static analysis.

In Fig. 11, ${A}_{x}$, ${A}_{y}$, and ${A}_{z}$ represent the force in x, y, and z direction corresponding to the path 1, respectively. ${B}_{x}$, ${B}_{y}$, and ${B}_{z}$ represent the force in x, y, and z direction corresponding to the path 2, respectively. Also, ${C}_{x}$, ${C}_{y}$, and ${C}_{z}$ represent the force in x, y, and z direction corresponding to the path 3, respectively. ${F}_{s}$ represents the disturbance force to excite the plate structure. The equilibrium equations of force on each path are established in Eq. (28).

28 $$\Sigma \overrightarrow{F}=0; \left({A}_{x}i+{A}_{y}j+{A}_{z}k\right)+\left({B}_{x}i+{B}_{y}j+{B}_{z}k\right)+\left({C}_{x}i+{C}_{y}j+{C}_{z}k\right)-{F}_{s}=0$$

The moment equations on each path are defined as Eq. (29).

$$\Sigma \overrightarrow{{M}_{O}}=0; \overrightarrow{{r}_{A}}\times \overrightarrow{{F}_{A}}+\overrightarrow{{r}_{B}}\times \overrightarrow{{F}_{B}}+\overrightarrow{{r}_{C}}\times \overrightarrow{{F}_{C}}+\overrightarrow{{r}_{s}}\times \overrightarrow{{F}_{s}}=0$$

29 $$\left|\begin{array}{ccc}i& j& k\\ {l}_{1}& -{l}_{f2}& 0\\ {A}_{x}& {A}_{y}& {A}_{z}\end{array}\right|+\left|\begin{array}{ccc}i& j& k\\ {-l}_{2}& {-l}_{f2}& 0\\ {B}_{x}& {B}_{y}& {B}_{z}\end{array}\right|+\left|\begin{array}{ccc}i& j& k\\ 0& {l}_{f1}& 0\\ {C}_{x}& {C}_{y}& {C}_{z}\end{array}\right|+\left|\begin{array}{ccc}i& j& k\\ -{d}_{1}& -{d}_{2}& 0\\ 0& 0& -{F}_{s}\end{array}\right|=0$$

**Figure 12:**
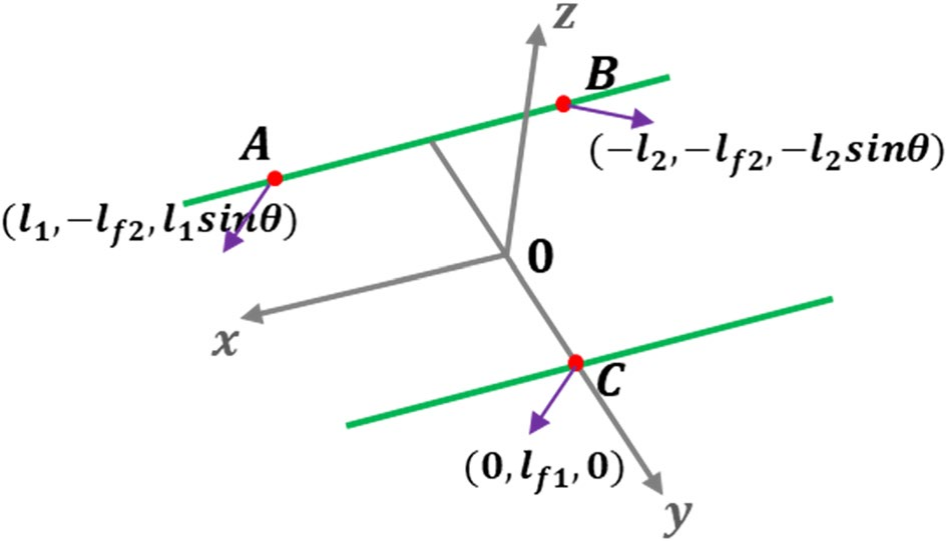
Displacement changes at three paths.

Based on the Fig.12, distance from actuator 1, actuator 2 and rubber mount to the center of gravity can be expressed in Eq. (30).

30 $$\begin{gathered} \sqrt {\left( {x_{A} + l_{1} } \right)^{2} + \left( {y_{A} - l_{f2} } \right)^{2} + \left( {z_{A} + l_{1} sin\theta } \right)^{2} } = \sqrt {l_{1}^{2} + l_{f2}^{2} } \hfill \\ \sqrt {\left( {x_{B} - l_{2} } \right)^{2} + \left( {y_{B} - l_{f2} } \right)^{2} + \left( {z_{B} - l_{2} sin\theta } \right)^{2} } = \sqrt {l_{2}^{2} + l_{f2}^{2} } \hfill \\ \sqrt {\left( {x_{C} } \right)^{2} + \left( {y_{C} - l_{f1} } \right)^{2} + \left( {z_{C} } \right)^{2} } = \sqrt {l_{f1}^{2} } \hfill \\ \end{gathered}$$

In order to compare the result of the static analysis with that of the dynamic analysis, the same boundary condition is applied to both analyses. Therefore, the forces in the x and y-directions are ignored. Eq. (31) can provide the necessary force for each path.

31 $$\begin{gathered} A_{z} = F_{1} = \theta k_{AZ} l_{1} \hfill \\ B_{z} = F_{2} = - \frac{{F_{s} \left( { - d_{1} l_{f1} - l_{2} l_{f1} - l_{2} d_{2} + l_{1} l_{f2} } \right) + \theta k_{AZ} l_{1} \left( { - l_{1} d_{2} + l_{2} d_{2} + l_{1} l_{f2} + d_{2} l_{2} } \right)}}{{2l_{f1} l_{2} }} \hfill \\ C_{z} = F_{NR} = - \frac{{F_{s} \left( {d_{1} l_{f1} - l_{2} l_{f1} + l_{2} d_{2} - l_{1} l_{f2} } \right) + \theta k_{AZ} l_{1} \left( { - l_{1} l_{f2} - l_{2} l_{f2} + l_{1} l_{f1} + l_{2} l_{f1} } \right)}}{{2l_{f1} l_{2} }} \hfill \\ \end{gathered}$$

To obtain the minimum force, partial derivative for needed force and the result is shown in Eq. (32).

32 $$\frac{2\theta {k}_{AZ}{l}_{1}\left({d}_{2}-{l}_{f2}\right)-\theta {k}_{AZ}{l}_{2}\left({d}_{2}+{l}_{f2}\right)}{2{{l}_{f1}l}_{2}}=0\Rightarrow {l}_{1}=\frac{{d}_{2}+{l}_{f2}}{2({d}_{2}-{l}_{f2})}{l}_{2}$$

By letting ${k}_{s}=\frac{{d}_{2}+{l}_{f2}}{2({d}_{2}-{l}_{f2})}$, the Eq. (32) can be rewritten as ${l}_{1}={k}_{s}{l}_{2}$. According to Table 1, ${l}_{f2}$ is set to 60 mm. To compare with the dynamic analysis, the ${k}_{s}$ is calculated to consider the position of shaker, ${d}_{2},$ which is changed from 5 to 100 mm.

**Figure 13:**
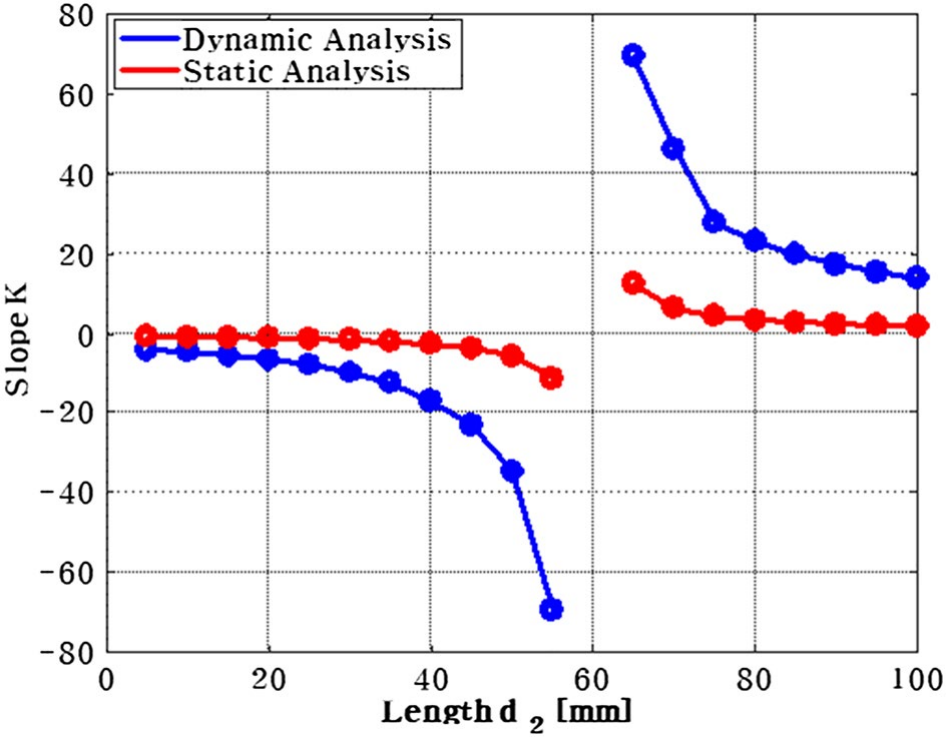
Comparison of static analysis and dynamic analysis (*generated with MATLAB R2016b,* https://mathworks.com).

The comparison result between static and dynamic analysis is shown in Fig. 13. The blue and orange line represent the result corresponding to dynamic and static, respectively. The results show a similar tendency in terms of slope. Here, the relationship between ${k}_{d}$ and ${k}_{s}$ should be considered through numerical analysis, and it is expressed in Eq. (33).

33 $${k}_{d}=q\cdot {k}_{s} (q=6.6)$$

### Optimal location of actuators

**Figure 14:**
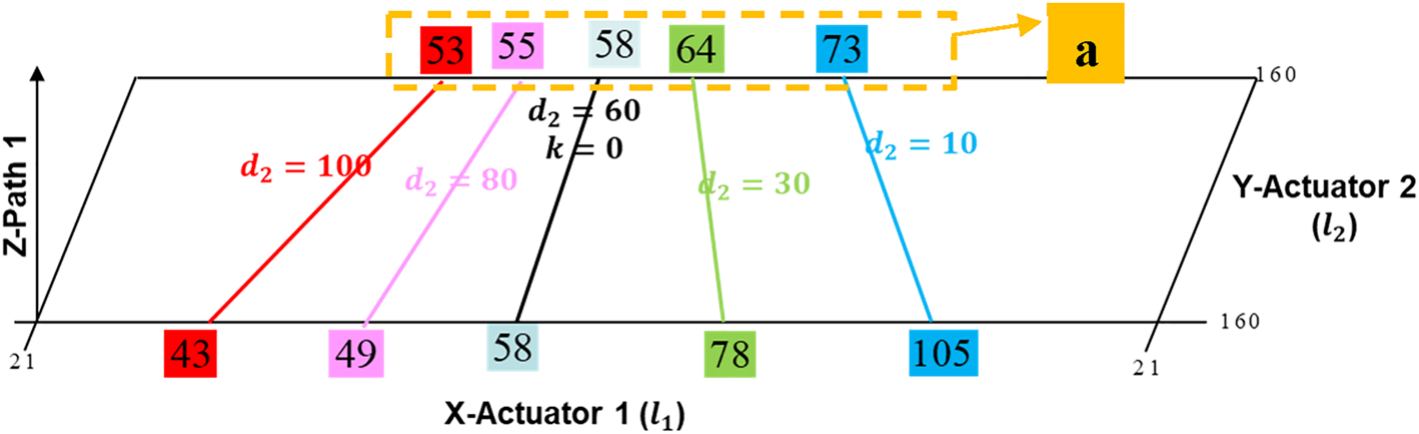
Schematic drawing of slope with the position of the shaker changes.

The results of Table 2 are represented in Fig. 14 so that the positional relationship between actuators 1, 2, and the shaker may be seen clearly.

The actuator 2 is positioned at 160 mm in Fig. 14, which indicates that it is at the edge's end. In Fig. 14, the slope is shown by the red, pink, black, green, and blue lines, which correspond to the minimized control force for the shaker position ${d}_{2}$. At that time, the location of shaker ${d}_{1}$ is fixed at 58 mm, and ${d}_{2}$ is changed to 10 mm, 30 mm, 60 mm, 80 mm, and 100 mm. Also, the optimized position of actuator 1 corresponding to actuator 2 is 73 mm, 64 mm, 58 mm, 55 mm, 53 mm, respectively, and it is defined as $a=[\mathrm{73,64,58,55,53}]$. In order to calculate the optimal position of actuator 1 through $a$ and ${d}_{2}$, the relationship between $a$ and ${d}_{2}$ is derived though curve fitting, and it is defined in Eq. (34).

34 $$a=p1\cdot {\left({d}_{2}\right)}^{3}+p2\cdot {\left({d}_{2}\right)}^{2}+p3\cdot {d}_{2}+p4$$

Through the curve fitting, the parameter ${p}_{1}$, ${p}_{2}$, ${p}_{3}$, and ${p}_{4}$ is determined to − 0.00003183, 0.007503, − 0.6944, and 79.15, respectively. Finally, the equation between the position of actuator 1 and actuator 2 can be written as Eq. (35).

35 $$y=q\times \frac{{d}_{2}+{l}_{f2}}{2\times \left({d}_{2}-{l}_{f2}\right)}\left(\mathrm{x}-\mathrm{a}\right)+\mathrm{P}$$

When the location of actuator 2 is known, the ideal position of actuator 1 may be determined using Eq. (35), and it is listed in Table 3. The best position of actuator 1 may be determined to be 101.279 mm, 85.695 mm, and 76.171 mm, respectively, when the actuator 2 is placed at 29 mm, 101 mm, and 145 mm.

**Table 3:** Optimized placement of actuators.

Y-Actuator 2 (mm)	Optimal location of X-Actuator 1 (mm)
29	101.279
101	85.695
145	76.171

### Simulation validation

**Figure 15:**
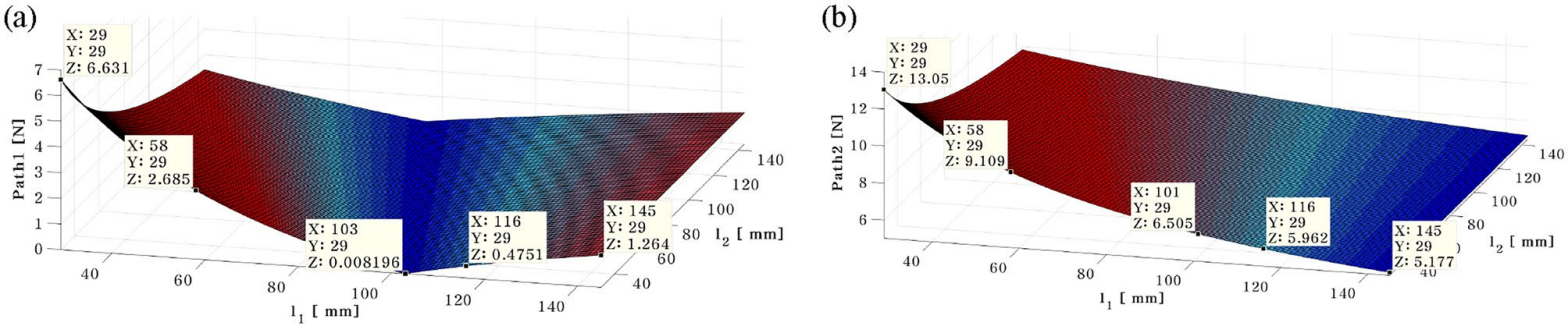
Needed control force on path 1 (**a**) and path 2 (**b**) when actuator 2 at 29mm (*generated with MATLAB R2016b*).

The dynamic analysis method is used to simulate in order to validate the actuator 1's optimal placement. Equation (26) can be used to determine the control forces acting on each actuator, when ${d}_{1}=58 \mathrm{mm}$, ${d}_{2}=10 \mathrm{mm}$, ${l}_{f1}=60 \mathrm{mm}$. Similar to Table 3, it can be separated into three cases based on the position of actuator 2 in this instance. First, the simulation results are displayed in Fig. 15 when actuator 2 is positioned at 29 mm, i.e., ${l}_{2}=29 \mathrm{mm}$.

The required force for paths 1 and 2 is shown in Fig. 15a,b, respectively. Additionally, Table 4 provides an overview of the simulation results for each position of Actuator 1. The results of the simulation show that if the actuator is shifted from the center of mass to the end plate, the required force decreases. Furthermore, when actuator 1 is positioned at 101 mm, the required force is less. When the location of actuator 1 is shifted from the near center of the plate to the end of the plate, independent of how the position of actuator 2 changes, the force required on path 2 is reduced, as shown in Fig. 15b. Given that the actuator 2 and shaker are adjacent, the actuator 2 requires more force than the actuator 1 does.

**Table 4:** Control force on the path 1 and path 2 when shaker is placed at 58 mm.

Shaker	Rubber Mount	Actuator 1 (mm)	Path1 [N]	Actuator 2	Path2 [N]
58 mm	60 mm	29	6.63	29 mm	13.05
		43	4.33		10.75
		58	2.69		9.10
		72	1.59		8.02
		87	0.71		7.13
		101	0.07		6.51
		116	0.48		5.96
		130	0.89		5.55
		145	1.26		5.18

**Figure 16:**
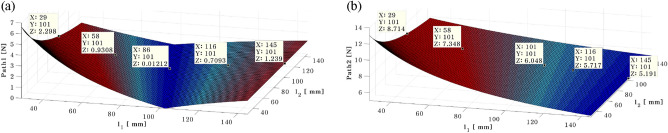
Needed control force on path 1 (**a**) and path 2 (**b**) when actuator 2 at 101mm (*generated with MATLAB R2016b*).

Second, when actuator 2 is positioned at 101 mm, or when the simulation results are depicted in Fig. 16.

**Figure 17:**
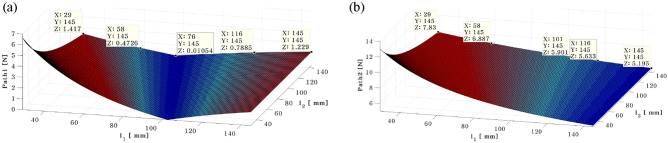
Needed control force on path 1 (a) and path 2 (b) when actuator 2 at 145mm (*generated with MATLAB R2016b).*

The required force for paths 1 and 2 is shown in Fig. 16a,b, respectively. Additionally, Table 5 provides a summary of the control force for each position of Actuator 1. As a result, the control force decreases as actuator 1 gets closer to the plate’s edge. When actuator 1 is placed at 87 mm, the control force is less. The control force increases as the actuator 1 moves away from 87 mm. Finally, the simulation results are displayed in Fig. 17 when actuator 2 is positioned at 145 mm, i.e., ${l}_{2}=145 \mathrm{mm}$.

**Table 5:** Control force on the path 1 and path 2 when shaker is placed at 58 mm.

Shaker	Rubber Mount	Actuator 1 (mm)	Path1 [N]	Actuator 2	Path2 [N]
58 mm	60 mm	29	2.30	101 mm	8.71
		43	1.57		7.99
		58	0.93		7.35
		72	0.43		6.85
		87	0.02		6.41
		101	0.38		6.05
		116	0.71		5.72
		130	0.98		5.44
		145	1.23		5.19

The required force for paths 1 and 2 is shown in Fig. 17a,b, respectively. Additionally, Table 6 provides a summary of the control force for each position of Actuator 1. As a result, the control force decreases as actuator 1 gets closer to the plate's edge. When actuator 1 is placed at 72 mm, the control force is less. The control force is increased if the actuator 1 moves away from the 72mm. The results of the three case simulations are combined in Table 3 to validate the actuator placement that was optimized. The proposed dynamic technique can determine the optimal placement of the actuator using the aforementioned results.

**Table 6:** Control force on the path 1 and path 2 when shaker is placed at 58 mm.

Shaker	Rubber Mount	Actuator 1 (mm)	Path1 [N]	Actuator 2	Path2 [N]
58 mm	60 mm	29	1.42	145 mm	7.83
		43	0.93		7.34
		58	0.47		6.89
		72	0.11		6.52
		87	0.24		6.18
		101	0.52		5.90
		116	0.79		5.63
		130	1.01		5.41
		145	1.23		5.20

### Experimental validation of suggested formulation

#### Experiment design

**Figure 18:**
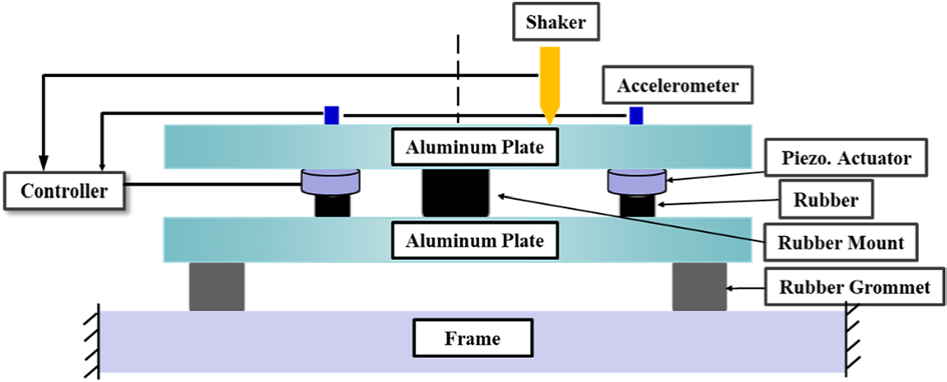
Schematic of the experiment.

**Figure 19:**
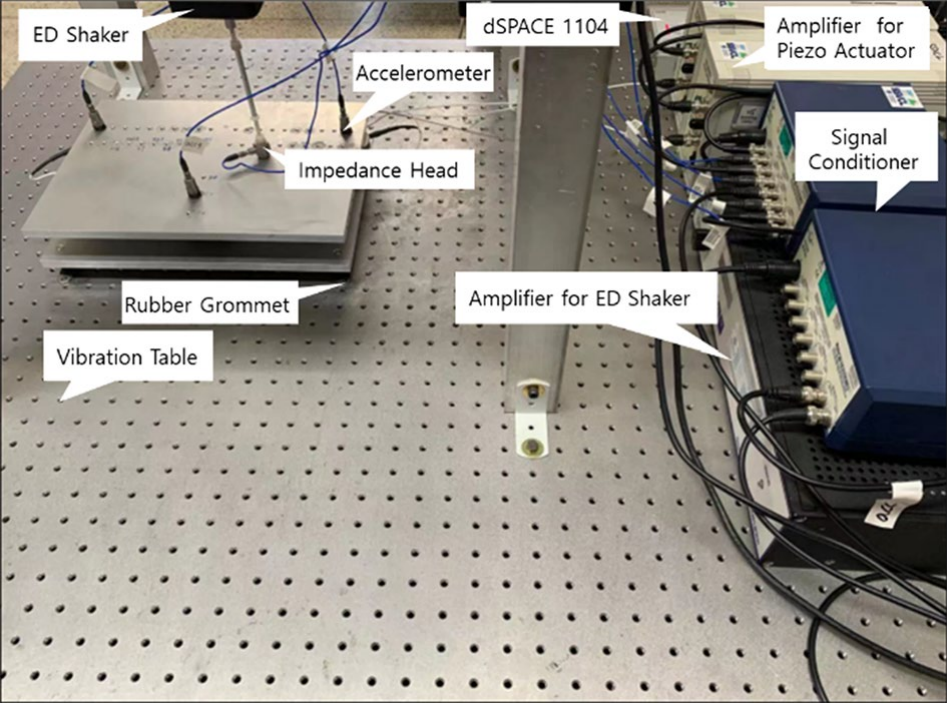
Experimental setup for active vibration control of plate.

To validate the results of the simulation, a feasibility experimental setup is created, and the design is shown in Fig. 18. Additionally, Fig. 19 displays the feasibility experimental setup. There are two aluminum plates in it. Between the aluminum plates are the passive and active paths. Two aluminum plates are 330 mm by 240 mm in size, with a 10 mm thickness. A rubber mount and a piezoelectric actuator make up the active path. Additionally, the passive path only has rubber mounts. An impedance head attached to the end of the stinger is used to measure the disturbance force, which is applied to the source component of the structure using a sinusoidal wave at 460 Hz in order to excite it. The signal is measured in real-time using the d-SPACE. The accelerometer signal that corresponds to the source component is employed as an actuator input signal using an adaptive filter to control the source part.

## Results and discussion

**Figure 20:**
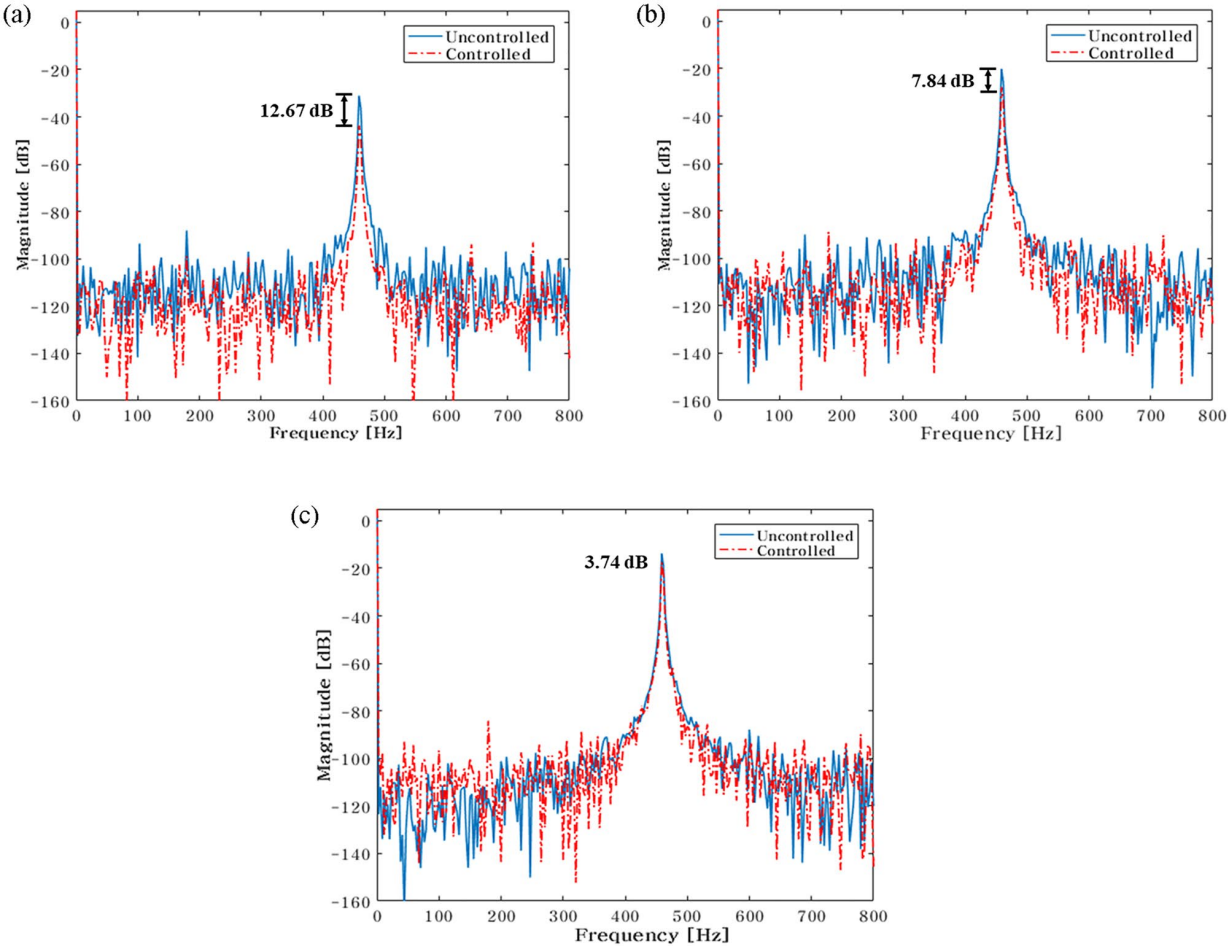
Comparison of measured accelerometer spectra for sinusoids: (**a**) Path 1 (active); (**b**) Path 2 (active); (**c**) Path 3 (passive). Key, blue solid line Uncontrolled; red dotted line Controlled.

The experiment is conducted utilizing a feasibility experimental setup to validate the simulation results. To conduct the experiment under the same circumstances as the simulation, the active and passive routes are included in the same simulation. Actuator 1 shifts to locations at 29mm, 43.5mm, 58mm, 72.5mm, 87mm, 101.5mm, 116mm, 130.5mm, and 145mm when the location is taken into account. Additionally, an actuator 2 is positioned at 29 mm, 101 mm, and 145 mm to carry out the experiment's three main scenarios. First, Fig. 20 summarizes the FFT results for the actuator 2 at a 29 mm location. Only the outcomes for the scenario when actuator 1 is positioned optimally are reported when the total experimental findings are done by FFT.

When actuator 1 is positioned at 101.5mm in Fig. 20, the blue line and red dot lines indicate the FFT results for uncontrolled and controlled, respectively. It represents the active path in Fig. 20a,b, with the vibration reduction impact being 12.67 dB and 7.84 dB, respectively. Additionally, it depicts the passive path in Fig. 20c, where the effect of vibration reduction is 3.74 dB. The actuator 2 is situated close to the shaker when conducting the experiment of the overall case, that is, placing the actuator 2 at 29mm, 101mm, and 145mm. As a result, path 2 reduces vibrations at a slower rate than path 1. The rubber mount is the only component of the passive path. As a result, the active path has a higher effect on vibration reduction.

**Figure 21:**
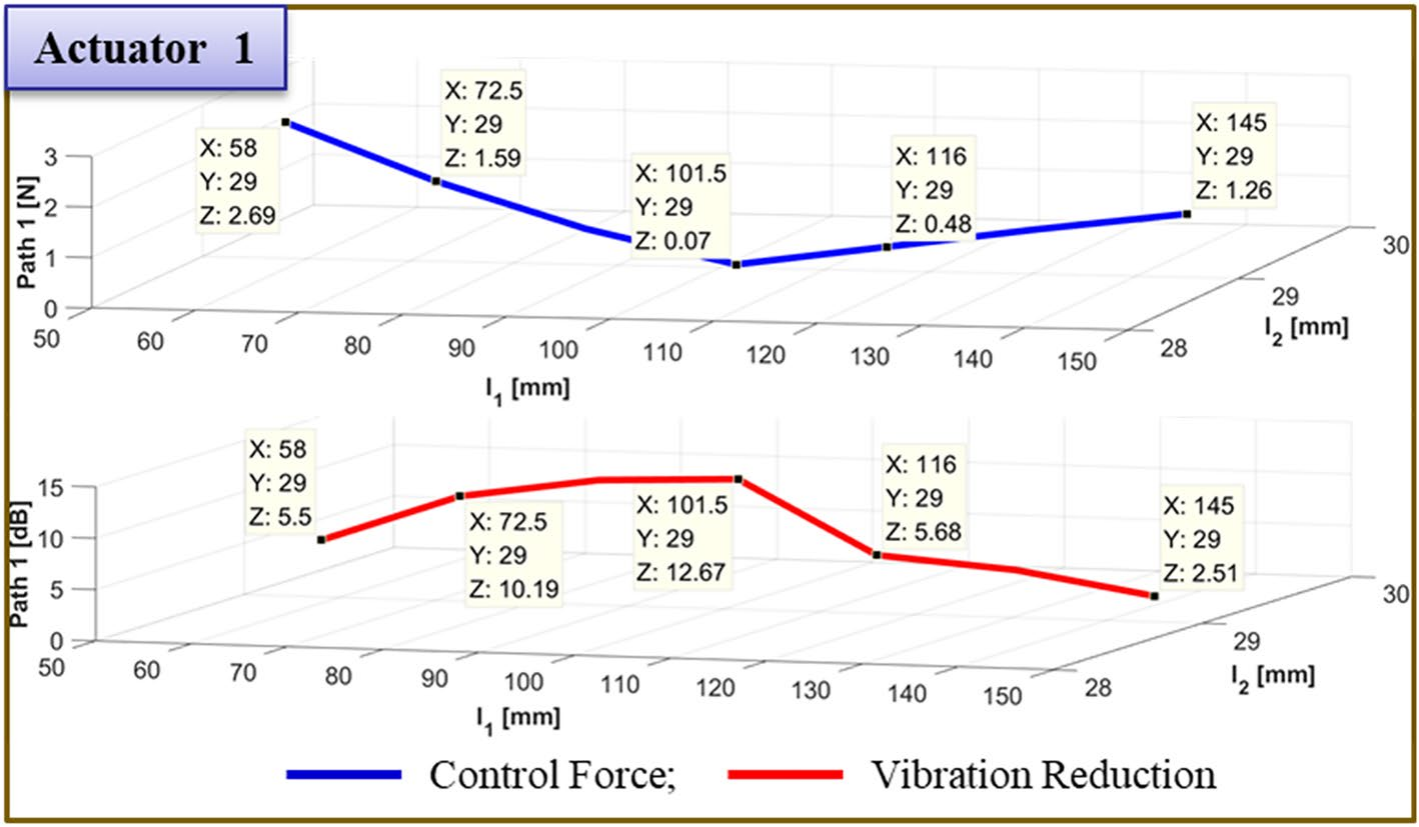
Comparison of simulations and experiment for sinusoid signals at actuator 1. Key, blue solid line Control Force; red solid line Vibration Reduction.

**Figure 22:**
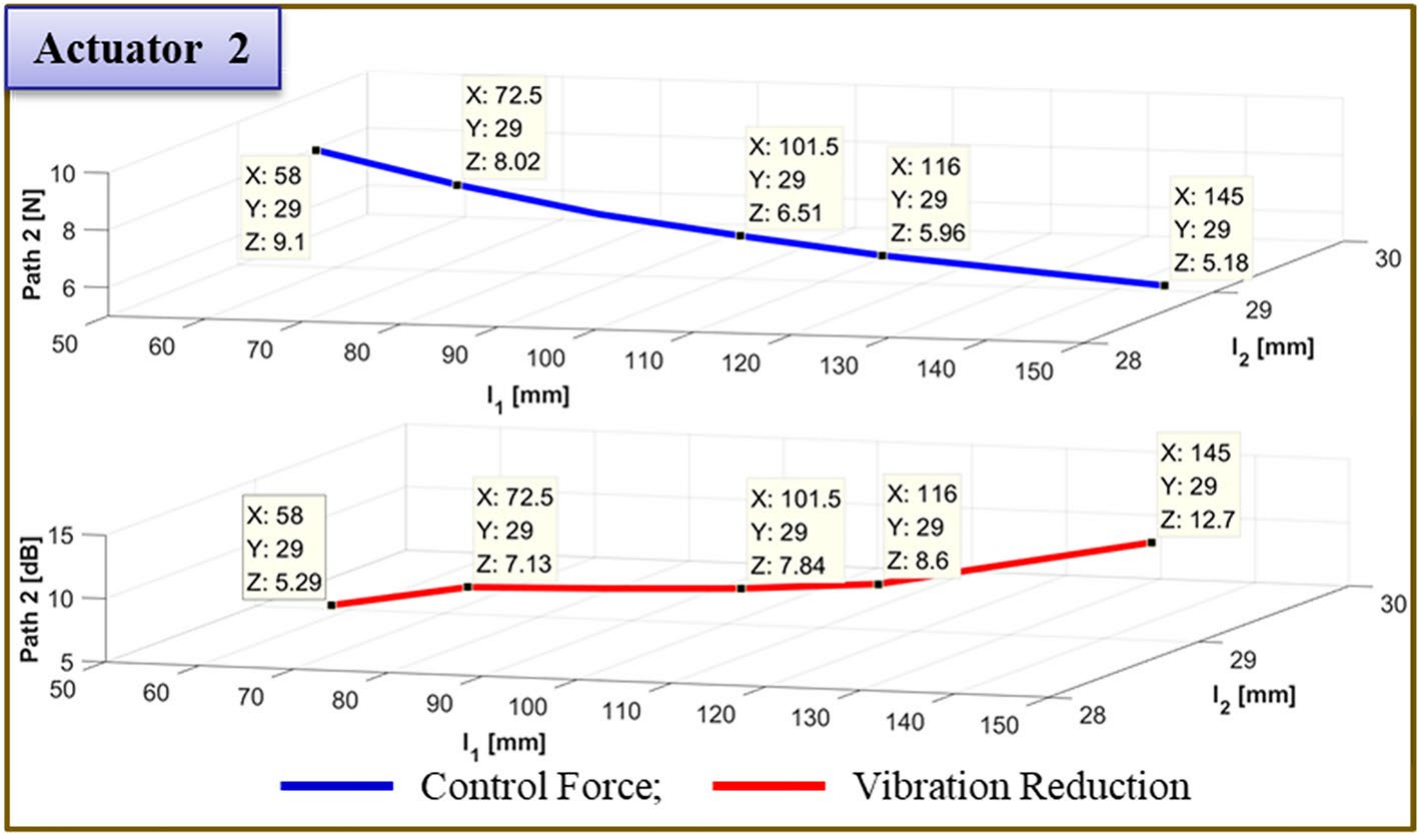
Comparison of simulations and experiment for sinusoid signals at actuator 2. Key, blue solid line Control Force; red solid line Vibration Reduction.

The results of moving actuator 1's position to test the effectiveness of vibration reduction are compiled in Table 7. Figs. 21 and 22 depict, respectively, the required control force and vibration reduction associated with changing the position of actuator 1. The control force is depicted in Figs. 21 and 22 by the blue line, which is determined by Eq. (26). The vibration reduction performance, which was determined through experimentation, is represented by the red line.

**Table 7:** Reduction of vibration of each path at 460Hz when actuator 1 moved from 58 to 145 mm.

Rubber Mount	Shaker	Actuator 2	Actuator 1 (mm)	Path 1 (Peak) [dB]	Path 2 (Peak) [dB]	Path3 (Peak) [dB]
60 mm	58 mm	29 mm	58.0	5.5↓	5.29↓	0.77↓
			72.5	10.19↓	7.13↓	3.26↓
			87.0	12.17↓	7.43↓	4.27↓
			101.5	**12.67↓**	**7.84↓**	**3.74↓**
			116.0	5.68↓	8.60↓	4.30↓
			130.5	*4.64↓*	*3.10↓*	*11.21↓*
			145.0	2.51↓	12.70↓	2.27↓

Significant values are in [bold and italics].

**Figure 23:**
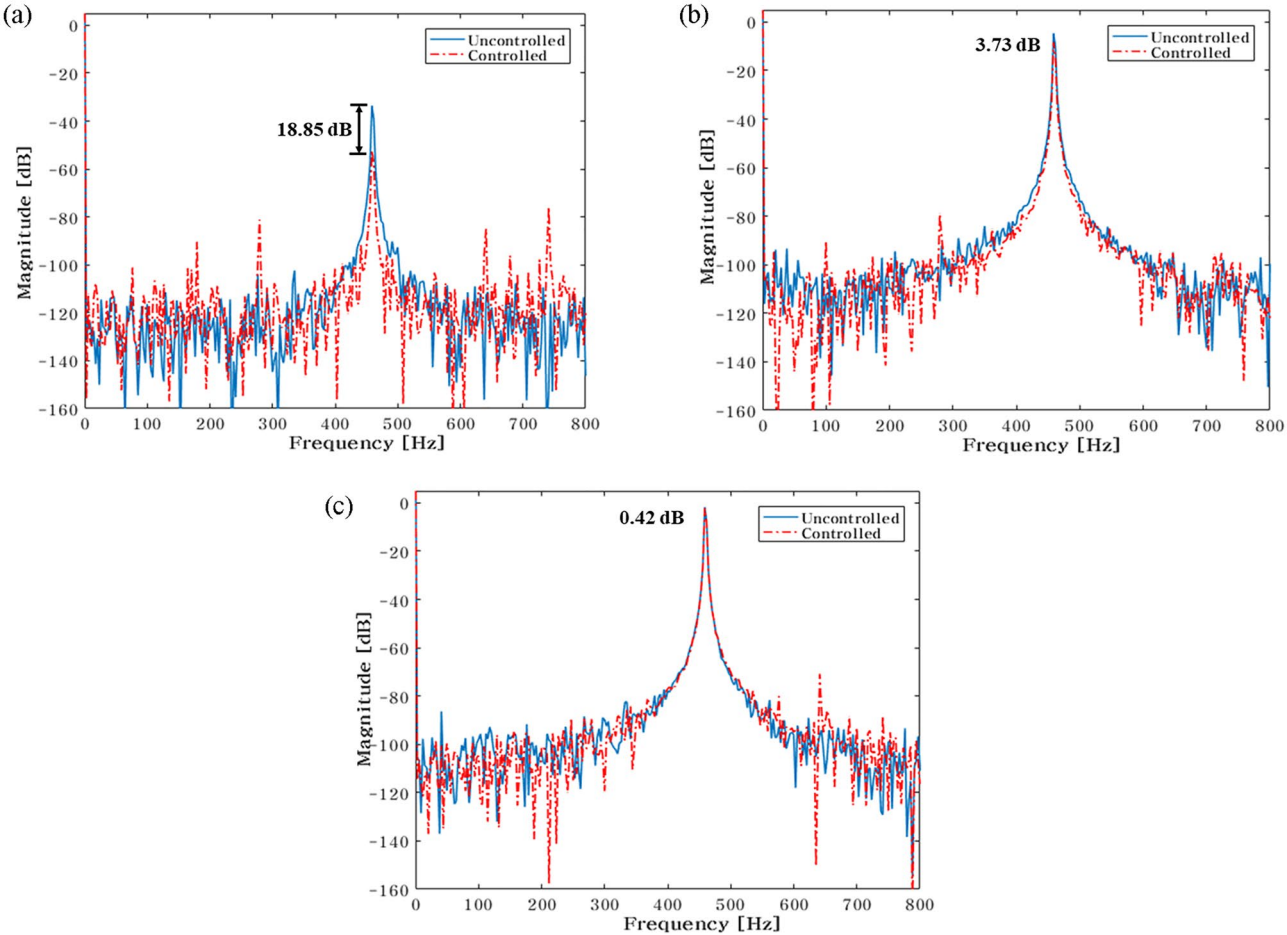
Comparison of measured accelerometer spectra for sinusoids: (**a**) Path 1 (active); (**b**) Path 2 (active); (**c**) Path 3 (passive). Key, blue solid line Uncontrolled; red dotted line Controlled.

The control force is less in the case of actuator 1, when it is placed at 101.5mm. The reduction in vibration is then larger than it is in other locations. Additionally, when actuator 2 is towards the ends of its edges, the control force has a smaller value. Second, the experiment's findings are compiled in Fig. 23 for the actuator 2’s position at 101.5 mm. In Fig. 23, the blue line and red dot line illustrate, respectively, the uncontrolled and regulated FFT results when actuator 1 is positioned at 87mm. It represents the active path in Fig. 23a,b, with the vibration reduction impact being 18.85 dB and 3.73 dB, respectively. Additionally, it depicts the passive path in Fig. 23c, where the effect of vibration reduction is 0.42 dB. The rubber mount is the only component of the passive path. As a result, the active path has a higher effect on vibration reduction.

**Figure 24:**
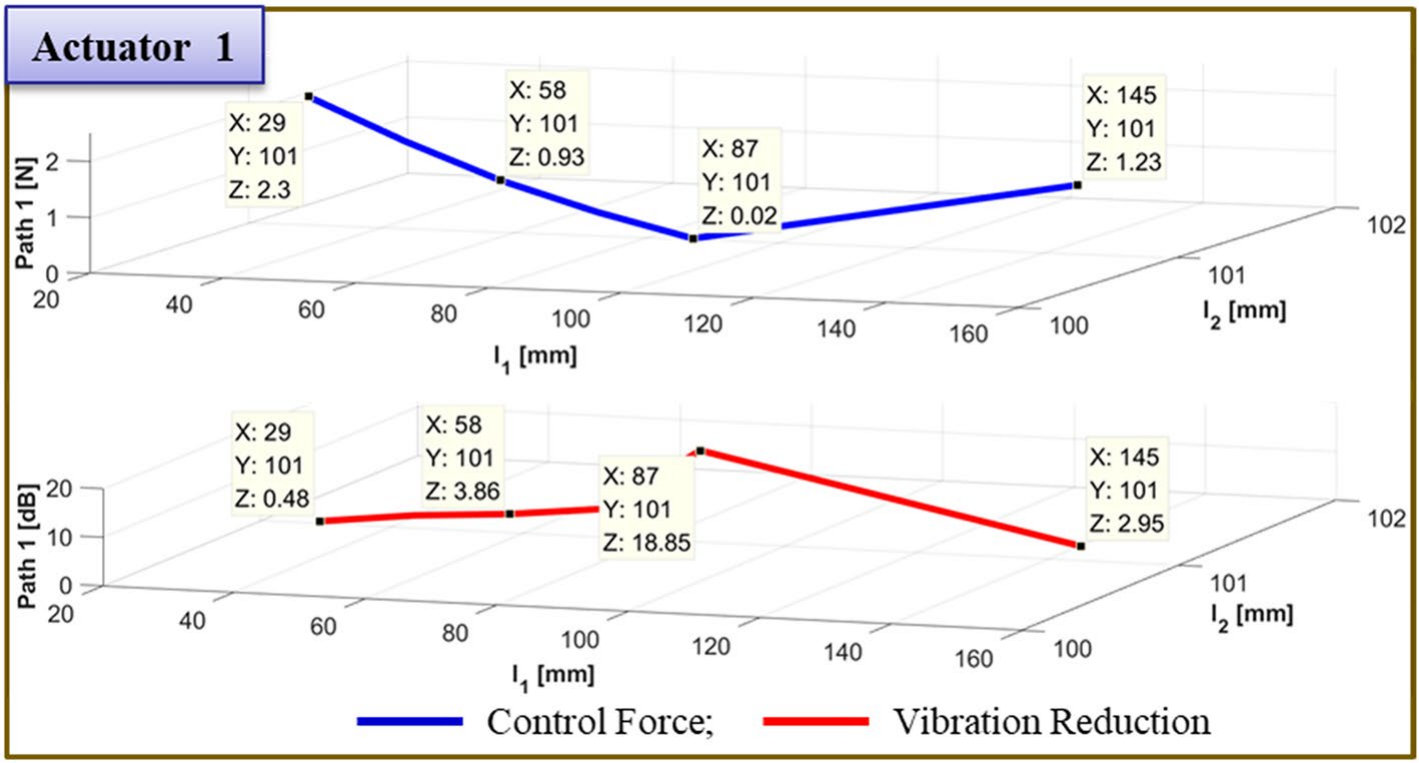
Comparison of simulations and experiment for sinusoid signals at actuator 1. Key, blue solid line Control Force; red solid line Vibration Reduction.

**Figure 25:**
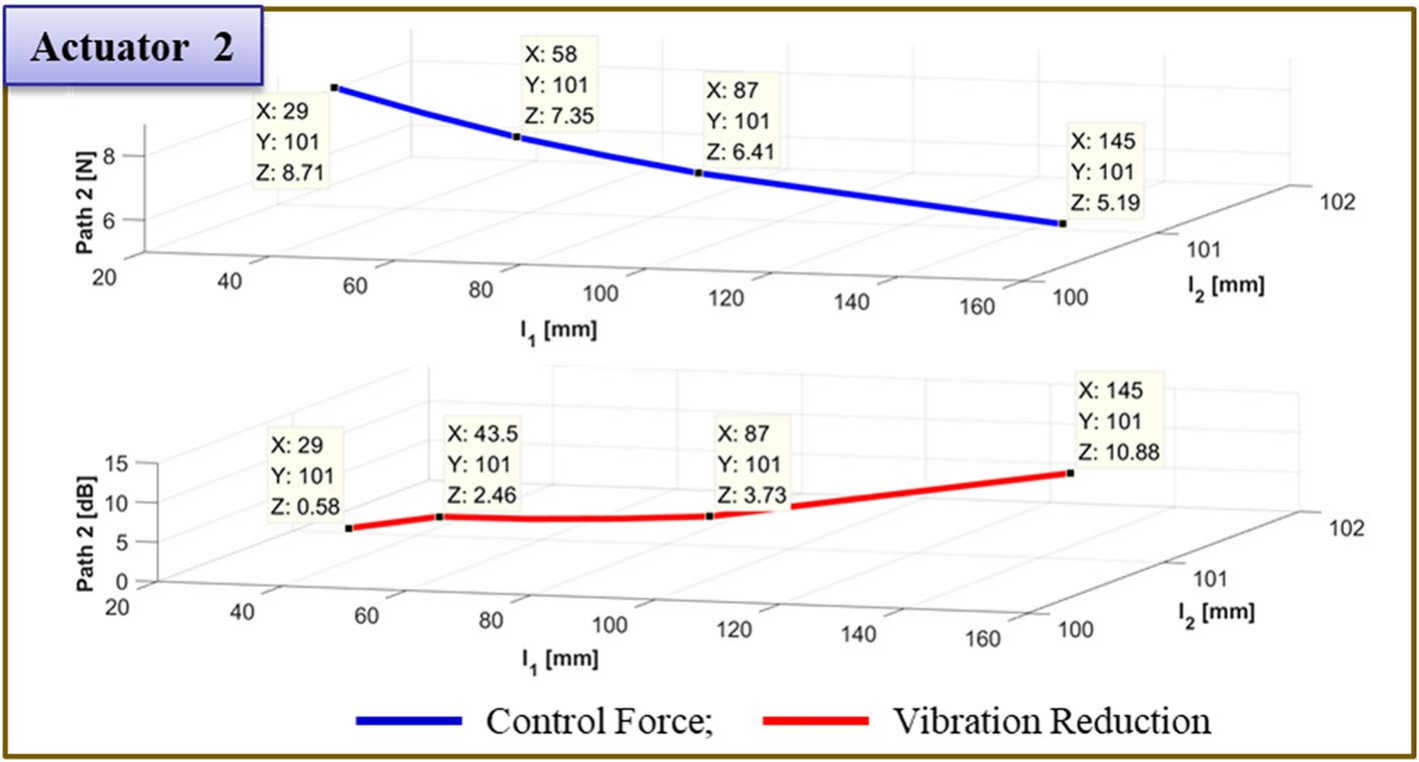
Comparison of simulations and experiment for sinusoid signals at actuator 2. Key, blue solid line Control Force; red solid line Vibration Reduction.

To verify the effectiveness of the vibration reduction, the actuator 1 position is modified. The findings are given in Table 8. Figs. 24 and 25 show, respectively, the required control force and vibration reduction performance related to adjusting the position of path 1. The control force in Figs. 24 and 25 is shown as a blue line that is derived from Eq. (26), and the vibration reduction effect is shown as a red line that is derived from experiment. When actuator 1 is positioned at 87 mm, the control force is lower, and the performance of vibration reduction is better than in other places. Additionally, when actuator 2 is towards the ends of its edges, the control force has a smaller value.

**Table 8:** Reduction of vibration of each path at 460Hz when actuator 1 moved from 29 to 145 mm.

Rubber Mount	Shaker	Actuator 2	Actuator 1 (mm)	Path 1 (Peak) [dB]	Path 2 (Peak) [dB]	Path3 (Peak) [dB]
60 mm	58 mm	101 mm	29.0	0.48↓	0.58↓	***0.38↑***
			43.5	2.64↓	2.46↓	0.61↓
			58.0	3.86↓	2.58↓	1.29↓
			72.5	5.88↓	3.03↓	0.91↓
			87.0	**18.85↓**	**3.73↓**	**0.42↓**
			101.5	6.01↓	8.14↓	2.90↓
			116.0	*1.73↓*	*8.98↓*	*1.00↓*
			130.5	*2.43↓*	*0.42↓*	*5.35↓*
			145.0	2.95↓	10.88↓	0.60↓

Significant values are in [bold, italics and bold italics].

**Figure 26:**
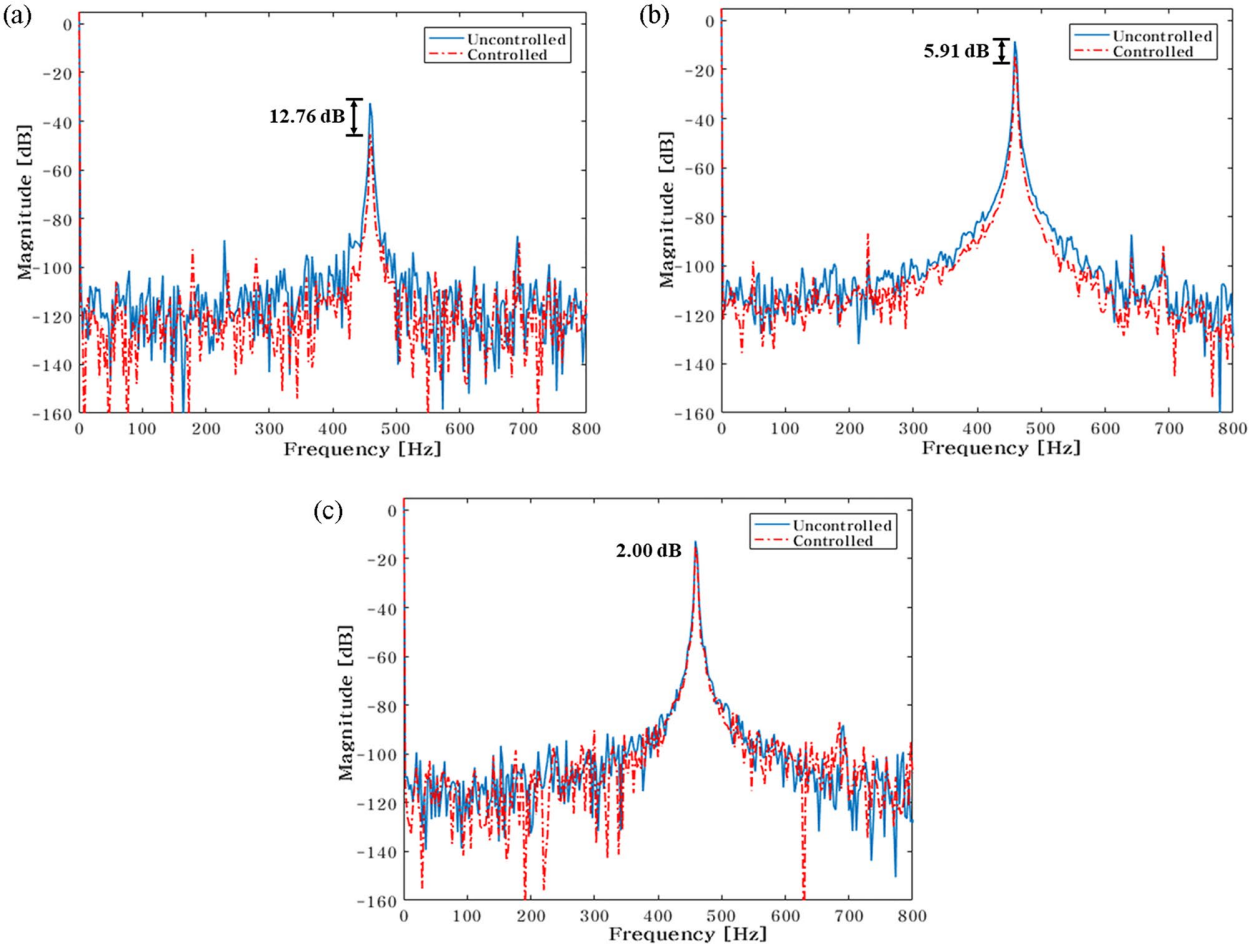
Comparison of measured accelerometer spectra for sinusoids: (**a**) Path 1 (active); (**b**) Path 2 (active); (**c**) Path 3 (passive) . Key, blue solid line Uncontrolled; red dotted line.

Finally, Fig. 26 provides a summary of the experiment's findings when the actuator 2 is positioned at 145 mm. When actuator 1 is positioned at 72.5mm in Fig. 26, the blue line and red dot lines indicate the FFT results for uncontrolled and controlled, respectively. It depicts the active path in Fig. 26a,b, and the effects of vibration reduction are 12.76 dB and 5.91 dB, respectively. Additionally, it depicts the passive path in Fig. 26c, and the effect of vibration reduction is 2 dB.

**Figure 27:**
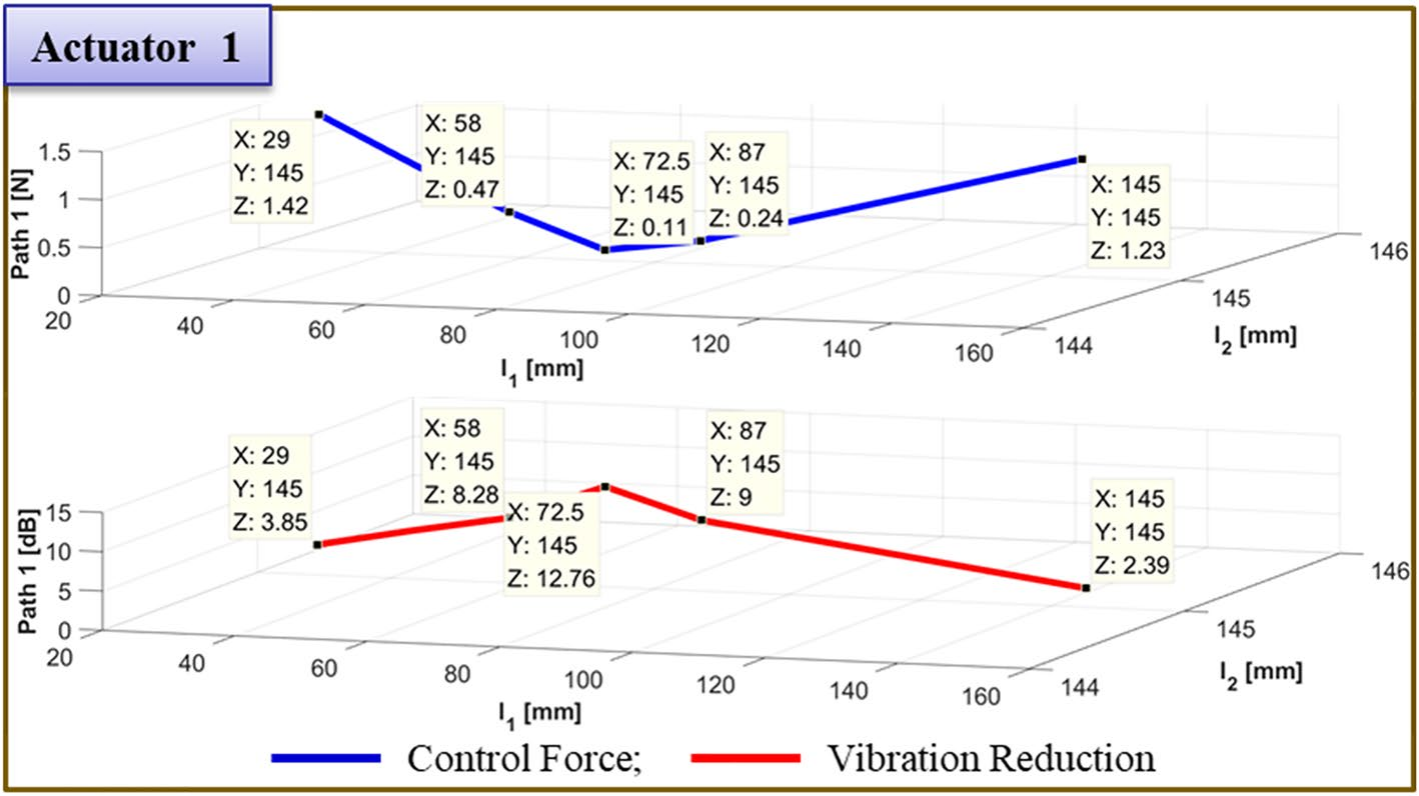
Comparison of simulations and experiment for sinusoid signals at actuator 1. Key, blue solid line Control Force; red solid line Vibration Reduction.

**Figure. 28:**
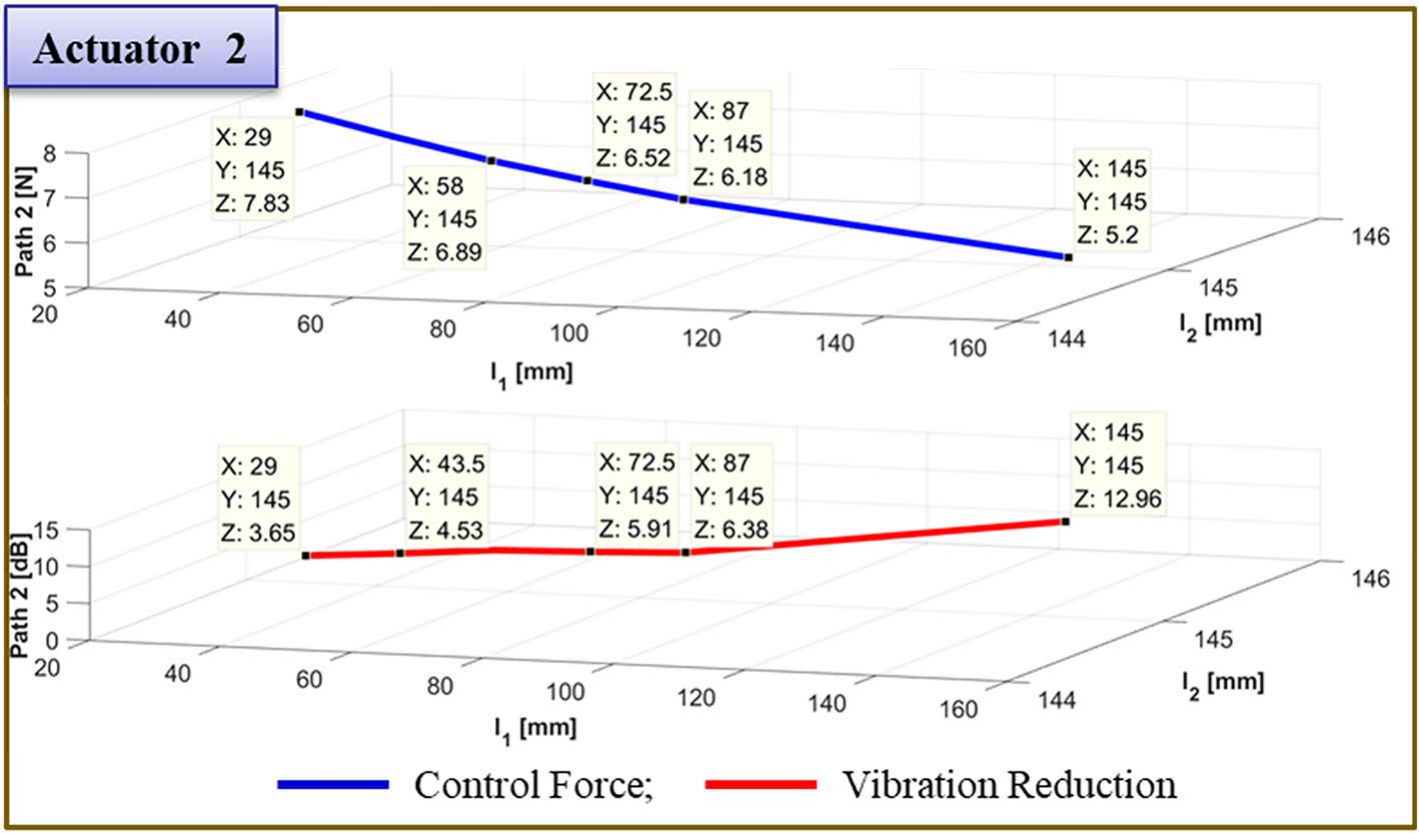
Comparison of simulations and experiment for sinusoid signals at actuator 2. Key, blue solid line Control Force; red solid line Vibration Reduction.

To verify the effectiveness of the vibration reduction, the actuator position is modified. The findings are given in Table 9. Figs. 27 and 28 show, respectively, the required control force and vibration reduction performance related to changing the position of actuator 1. The control force is depicted in Figs. 27 and 28, where it is determined using Eq. (26). The vibration reduction effect, as shown by the red line, was discovered through experimentation. The control force is less in the case of actuator 1, where it is placed at 72.5mm. The reduction in vibration is then larger than it is in other locations. Additionally, when actuator 2 is towards the ends of its edges, the control force has a smaller value.

**Table 9:** Reduction of vibration of each path at 460Hz when actuator 1 moved from 29 to 145 mm.

Rubber Mount	Shaker	Actuator 2	Actuator 1	Path 1 (Peak) [dB]	Path 2 (Peak) [dB]	Path3 (Peak) [dB]
60 mm	58 mm	145 mm	29.0mm	3.85↓	3.65↓	1.82↓
			43.5mm	6.16↓	4.53↓	2.22↓
			58.0mm	8.28↓	5.56↓	2.89↓
			72.5mm	**12.76↓**	**5.91↓**	**2.00↓**
			87.0mm	9.00↓	6.38↓	2.52↓
			101.5mm	6.55↓	***1.81↑***	0.35↓
			116.0mm	4.23↓	7.19↓	1.04↓
			130.5mm	3.23↓	8.52↓	1.31↓
			145.0mm	2.39↓	12.96↓	1.70↓

Significant values are in [bold and bold italics].

Comparing the simulated and experimental results reveals a fair degree of agreement. As a result, the experiment validates the proposed method, which uses a dynamic and static analysis. The suggested method can also be used to advise the active mounting system's optimal placement.

### Comparison with beam-type continuous smart structure

**Figure. 29:**
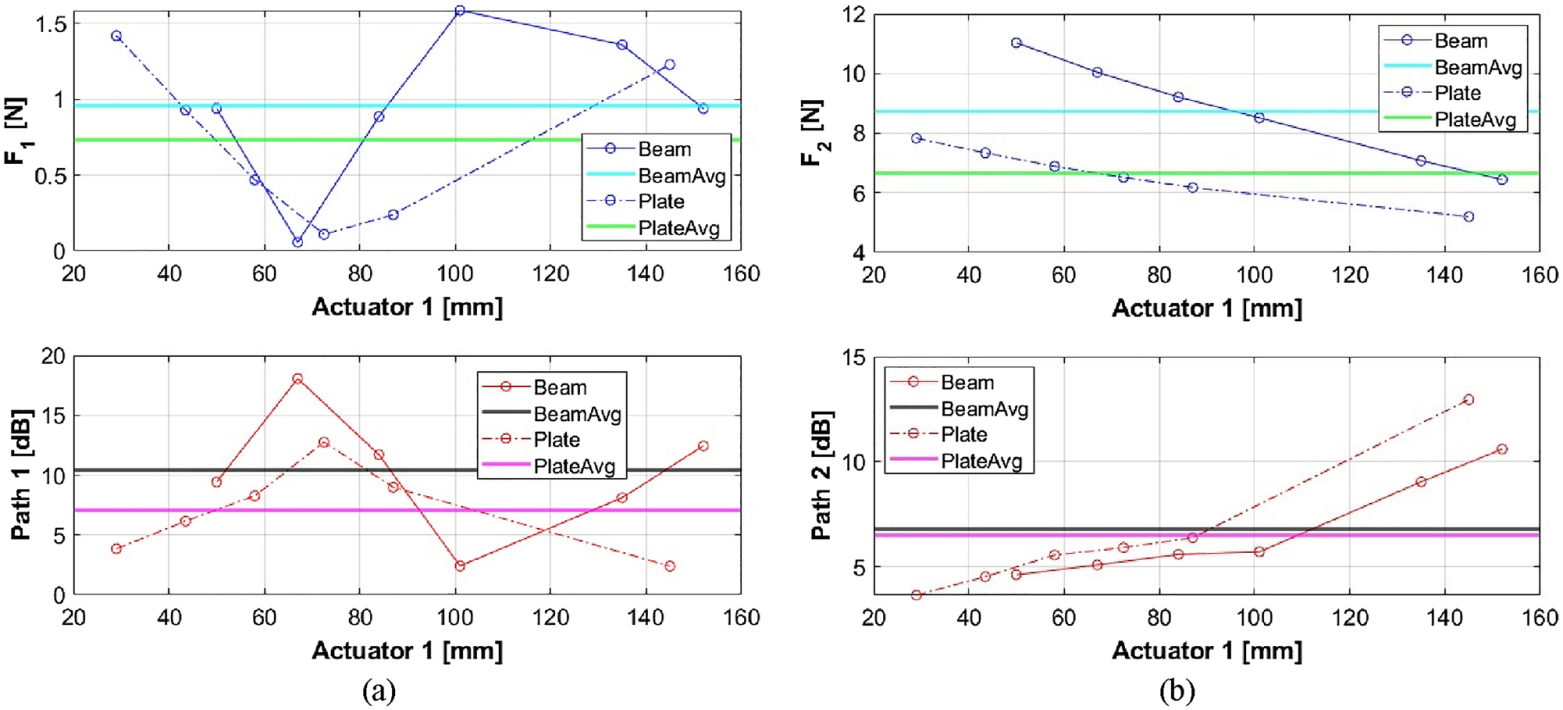
Comparison of beam and plate in (**a**) path 1 and (**b**) path 2.

As discussed in the first section, the same process was applied to a beam-type continuous smart structure and the optimized location of active paths are investigated and experimentally validated^33^. While the moment of inertia in the y-direction is only considered for the beam-type structure, for more realistic investigation, the moments of inertia for both x- and y-directions are taken into account to the non-aligned plate structure in this study. Although the placement criteria are hard to compare each other, force required to each path is worth to be compared because those two structures are similar except the width (length of y-direction). Based on the comparison of results from beam-type and plate-type structures in path 1 with a fixed location of actuator 2, as shown in Fig. 29a, it can be found that the required forces to the secondary paths in the case of beam are relatively larger than those in the case of plate. However, the level of vibration reduction in the case of beam is bigger than the level in the case of plate. Since the plate structure has one additional passive path, some portion of the disturbance (excitation from the shaker) can be transmitted to it so that the required force would be reduced to active paths and the reduction level is also smaller. Also, larger area of plate could make the source vibration in each path smaller. In path 2, similar tendency can be found as described in Fig. 29b.

## Conclusion and future research

This work presents a dynamic and static mathematical analysis to determine the optimal placement criterion for the active mounting system. The proposed method is validated through numerical simulation and the design of a feasibility experiment in terms of its ability to reduce vibrations at the desired frequency. The followings are the principal contributions of this research: (1) The proposed quantification approach calculates the force of an active path. (2) Using quantifiable force, a dynamic analysis is performed to determine the optimal location of the active mounting system. (3) The static analysis method is proposed in order to derive the optimal position with explicit formula and validate the dynamic analysis. (4) The feasibility experiment is conducted in order to validate the simulation results for the static and dynamic approaches. (5) This method can be employed generally by simplifying the object and applying relevant dimensions, not limited to specific systems. This research focuses on the appropriate mounting position for active systems. A dynamic and static study is suggested in order to determine the optimal placement. Using the quantification approach, the control force on the active path is estimated during the dynamic analysis, and it is determined that the site has a lower control force. In addition, a static analysis is suggested in order to validate the dynamic analysis. In consideration of the relationship between static and dynamic analysis, the optimal location formula is defined. The optimal placement is derived using a predefined methodology and validated using dynamic analysis. In addition, the feasibility experiment is conducted using the feasibility experiment setup to validate simulation results. It demonstrates that the findings of the numerical simulation and the feasibility experiment are in good agreement. The results are compared with those in the case of beam structure^33^ and it is shown that the required forces to the secondary paths in the case of beam are relatively larger than those in the case of plate. However, the level of vibration reduction in the case of beam is bigger than the level in the case of plate.

In the future, when a multi-frequency signal, such as an amplitude- or frequency-modulated signal, is utilized as an input signal in a structure, simulation and feasibility experiments will be conducted to determine the optimal position of the active mounting system. Also, a stochastic analysis needs to be conducted to compromise with the results from harmonic signals. In order to create a more realistic model, the constitutive relationships and nonlinearities should be considered and the mounting location in real time will also be incorporated into the scenario. In addition, the proposed should be compared to existing methods of position optimization under the same structure and conditions.

## Data Availability

The datasets used and/or analysed during the current study available from the corresponding author on reasonable request.
